# Supplementary material for: The admixed brushtail possum genome reveals invasion history in New Zealand and novel imprinted genes
Source: Nat Commun. 2023 Oct 17;14:6364. doi: 10.1038/s41467-023-41784-8 (PMC10582058; doi:10.1038/s41467-023-41784-8)
Supplement: Supplementary file 1 — Supplementary Information [file 41467_2023_41784_MOESM1_ESM.pdf]

Supplementary Information

The admixed brushtail possum genome reveals invasion history in New Zealand and novel imprinted genes

Bond et al.

This pdf file contains:

- Supplementary Figures 1-5
    - Supplementary Figure 1. Identification of Y-chromosome contigs.
    - Supplementary Figure 2. Gene expression changes associated with liver development and weaning.
    - Supplementary Figure 3. Allele-specific methylation sites associated with known imprinted genes.
    - Supplementary Figure 4. Allele-specific methylation sites correlate with monoallelic expression of neighbouring genes.
    - Supplementary Figure 5. Defining the *H19* imprint control region in possum.
- Supplementary Tables 1-5
    - Supplementary Table 1. Marsupial genome assemblies to date.
    - Supplementary Table 2. Lipocalin gene cluster on Chromosome 3.
    - Supplementary Table 3. Primers and PCR conditions used in this study.
    - Supplementary Table 4. Statistics and coverage of nanopore data.
    - Supplementary Table 5. Genomic position of SNPs used to determine mono-allelic expression.
- Supplementary References
- Uncropped gel images for Supplementary Figure 1

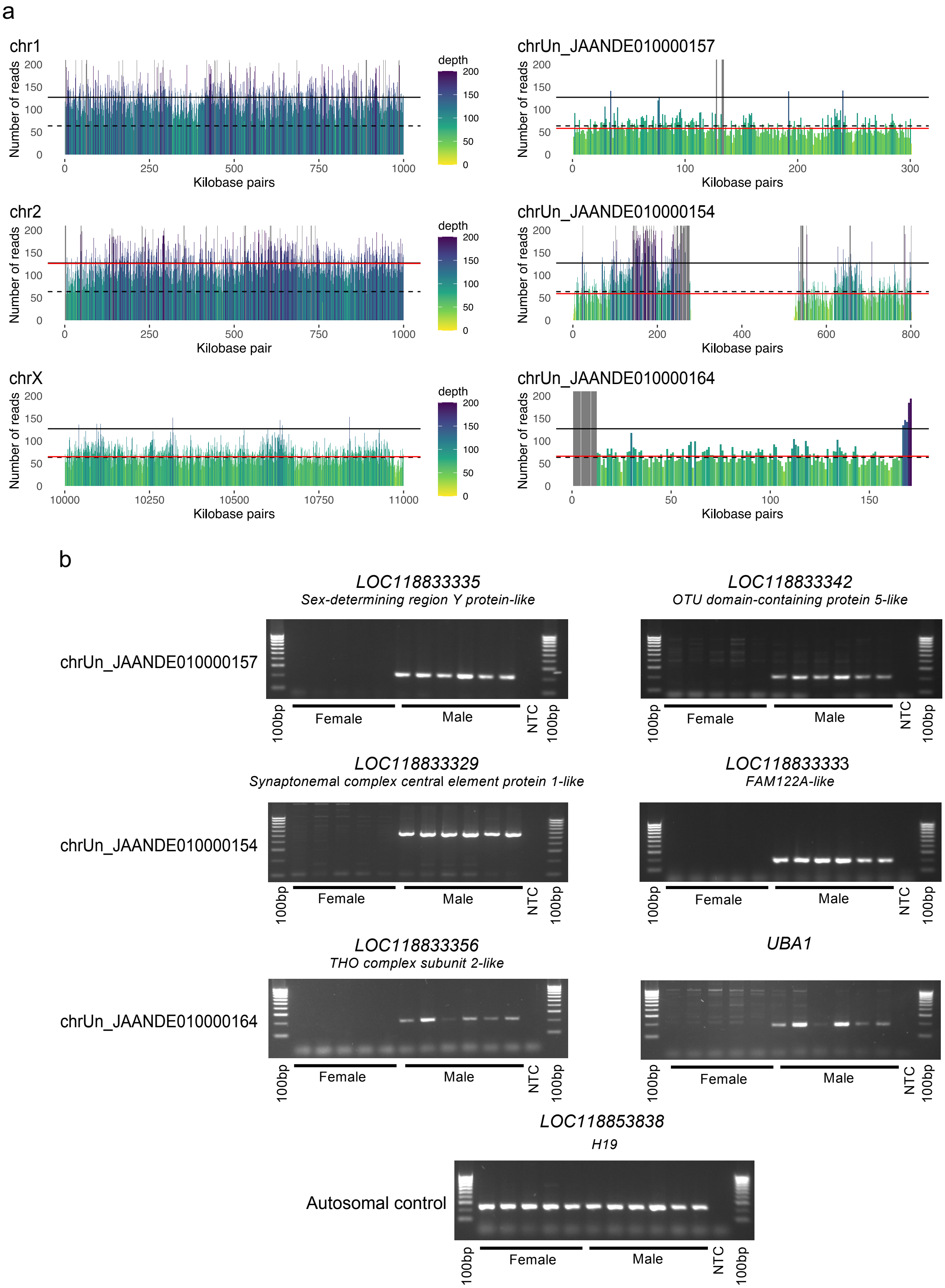

Supplementary Figure 1. Identification of Y-chromosome contigs. a) The number of sequencing reads mapped to 1 kilobase pair windows of known autosomal (chr1 and chr2), sex (chrX) and unassembled Y chromosome contigs (chrUn_JAANDE010000157, chrUn_JAANDE010000154 and chrUn_JAANDE010000164). As a reference, the solid black line represents the median number of reads mapped per kilobase pair for chr 1 and the dashed black line represents half of this value. The solid red line represents the median number of reads for each specific sequence chromosome/contig analysed. b) PCR amplification of *LOC118833335* (*Sex-determining region Y protein-like*), *LOC118833342* (*OTU domain-containing protein 5-like*), *LOC118833329* (*Synaptonemal complex central element protein 1-like*), *LOC118833333* (FAM122A-like), *LOC118833356* (*THO complex subunit 2-like*) and *UBA1* is specific to DNA from male possums. PCR amplification of *LOC118853838* (*H19*) was used as an autosomal control to confirm amplification from both male and female DNA samples. Primers and PCR conditions are given in Supplementary Table 3. 100bp represents the HyperLadder 100bp (Bioline) and NTC represents no template control.

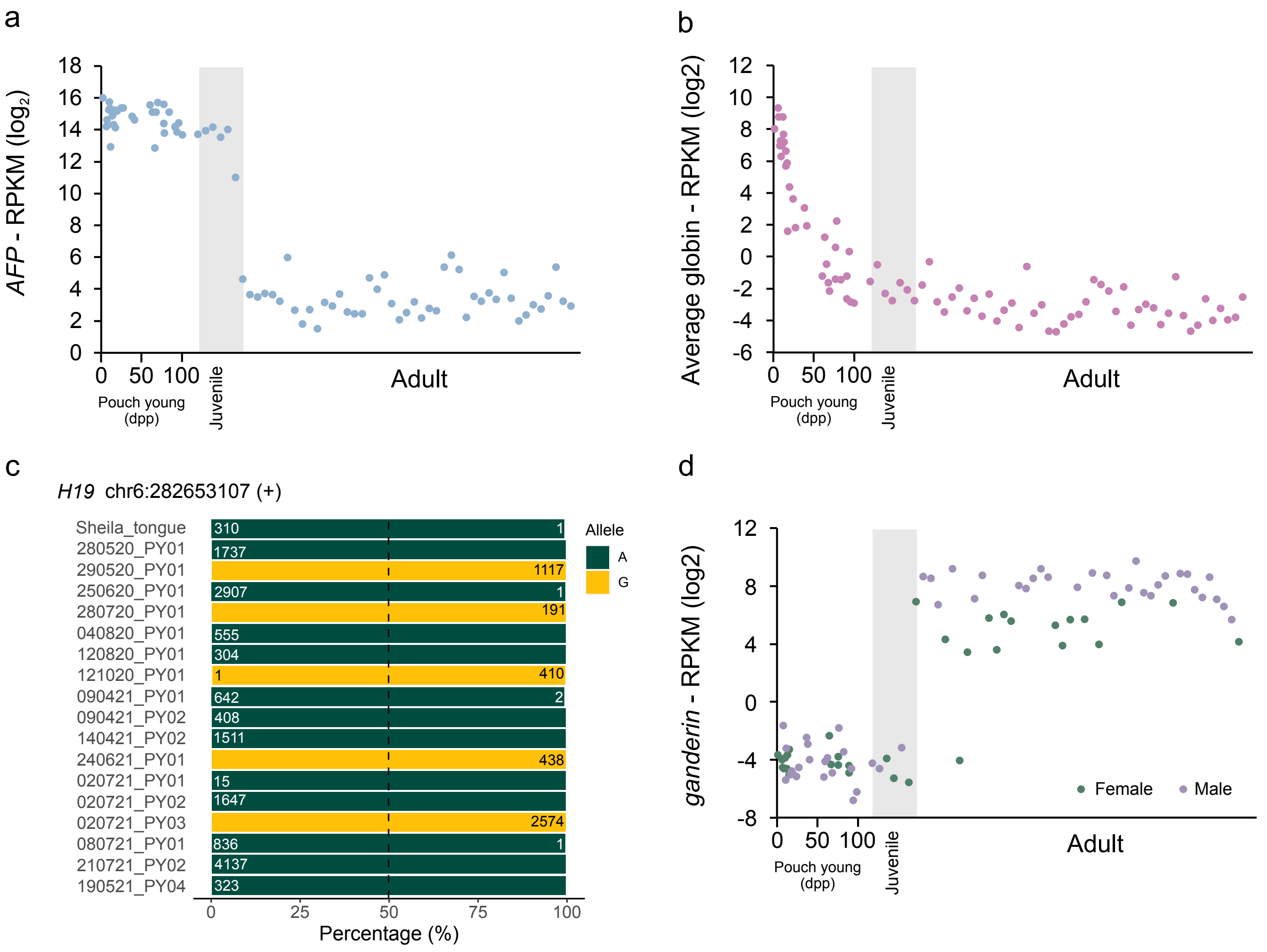

Supplementary Figure 2. Gene expression changes associated with liver development and weaning. Expression of (a) *AFP* (*LOC118852573*) and (b) globin genes in RNA-sequencing data of liver samples from pouch young, juvenile and adult possums. (c) Monoallelic expression of single nucleotide polymorphism within *H19* in RNA-sequencing data from heterozygous individuals. Values represent samples read depth (≥ 5). (d) Expression of *ganderin* (*LOC118843263*) in RNA-sequencing data of liver samples from pouch young, juvenile and adult possums. Source data are provided as a Source Data file.

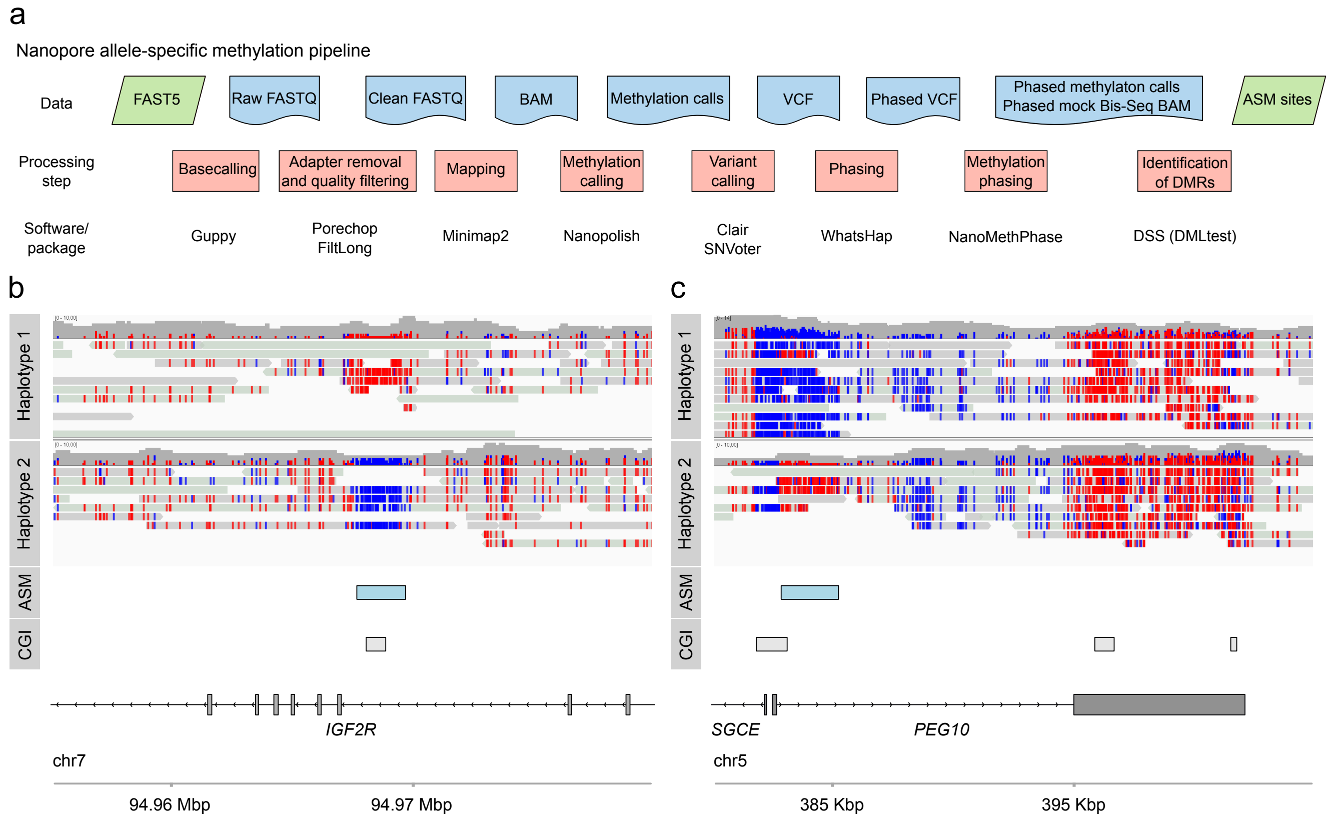

Supplementary Figure 3. Allele-specific methylation sites associated with known imprinted genes. Allele specific methylation discovery pipeline (a) and methylation haplotypes for *IGF2R* (b) and *PEG10* (c). Methylated cytosines are red, Unmethylated cytosines are blue; ASM, allele-specific methylation; CGI, CpG island.

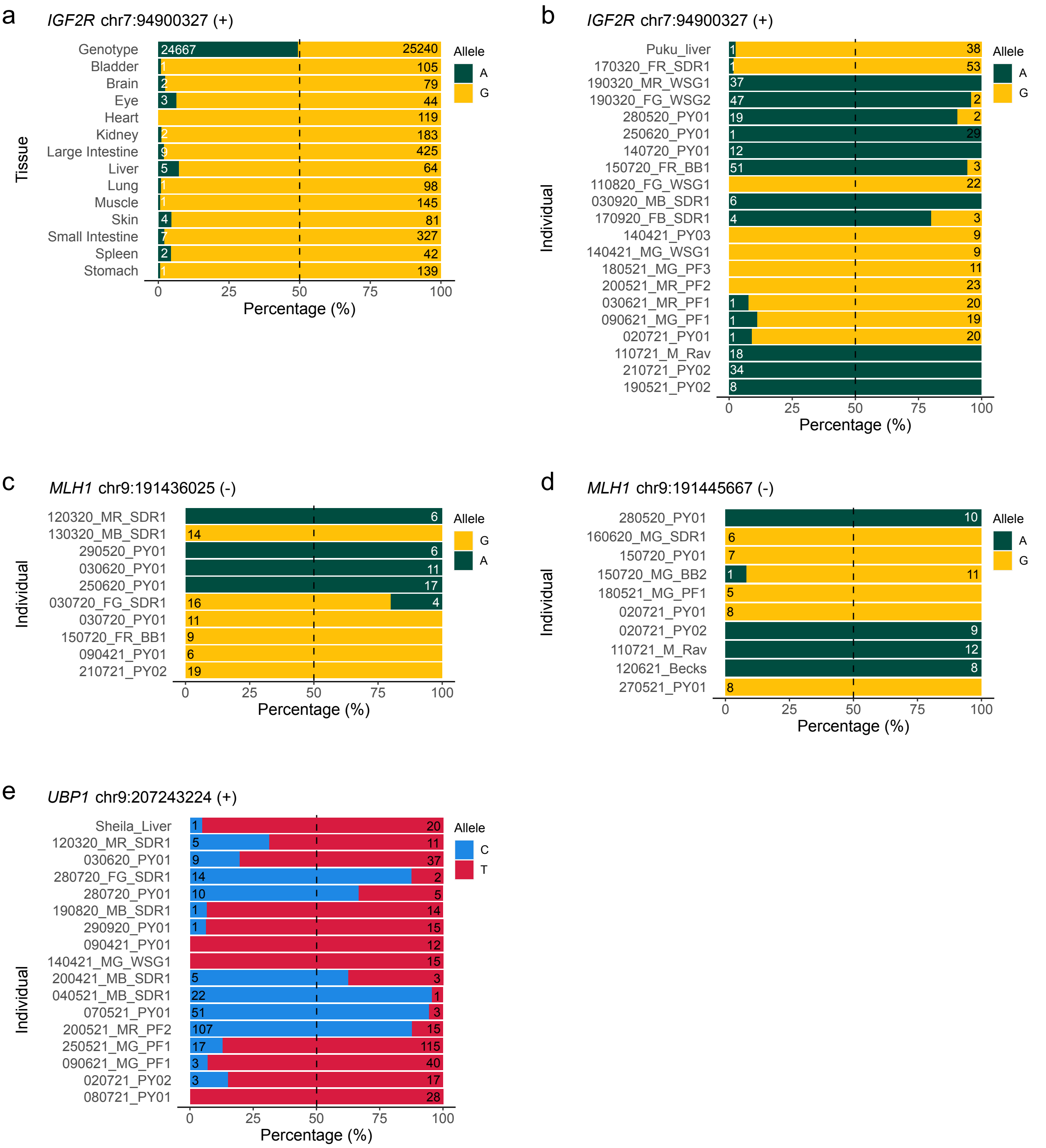

Supplementary Figure 4. Allele-specific methylation sites correlate with monoallelic expression of neighbouring genes. Monoallelic expression of single nucleotide polymorphisms within *IGF2R* in RNA-sequencing data from Sandy's tissues (a) and heterozygous individuals (b). Monoallelic expression of additional single nucleotide polymorphisms for *MLH1* (c and d) and *UBP1* (e) were analysed in heterozygous individuals. Values represent read depth (≥ 5). Source data are provided as a Source Data file.

a

b

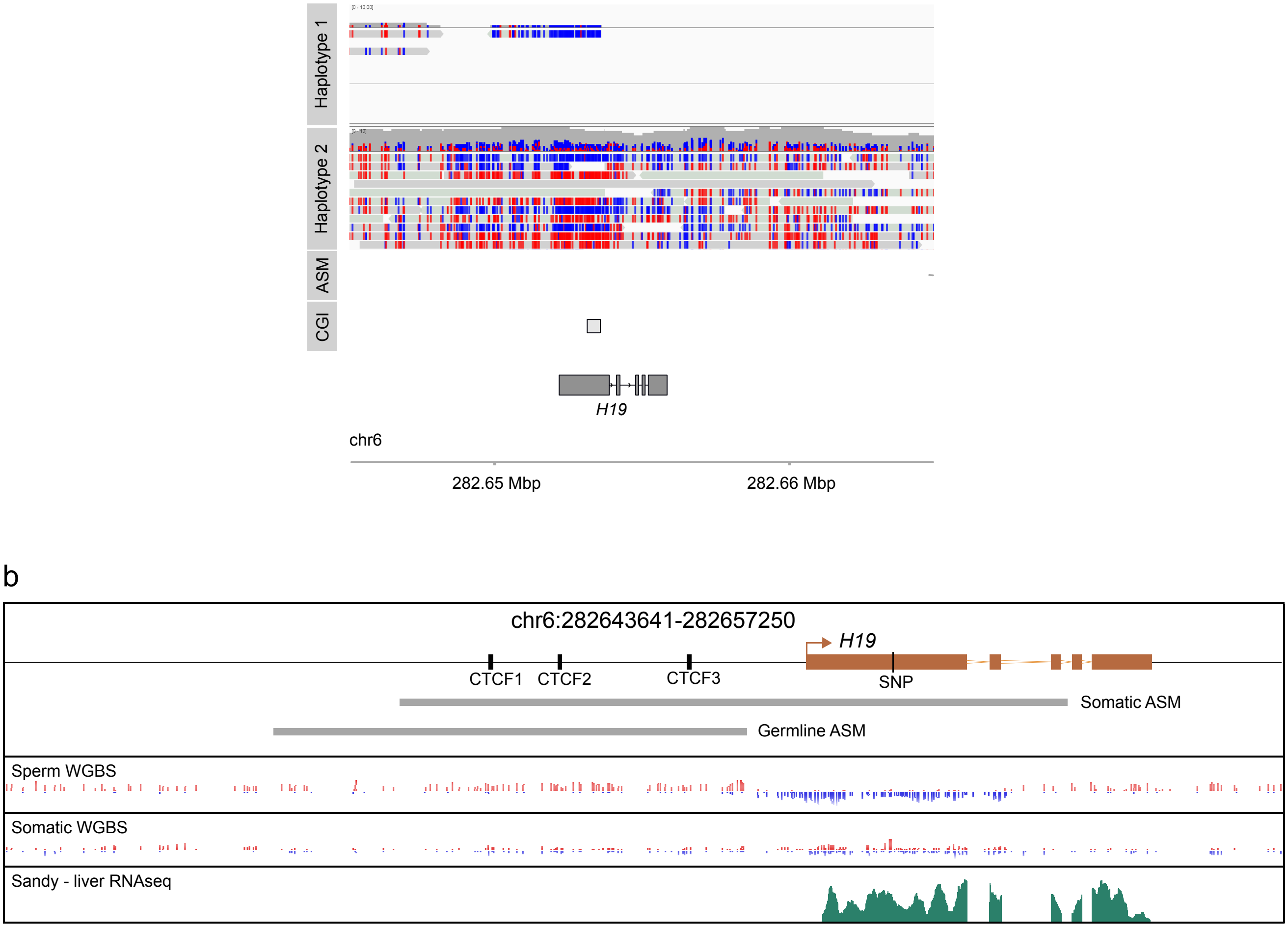

Supplementary Figure 5. Defining the *H19* imprint control region in possum. (a) Methylation haplotypes at the *H19* locus. No allele specific methylation site was defined for *H19* due to the lack of heterozygosity at this locus. However, within haplotype 2 there were reads with methylated cytosines (red) and reads with unmethylated cytosines (blue) covering region chr6:282647885-282654945 (defined as the somatic ASM) (b) Screenshot of WGBS reads from somatic non-germline cells from pouch young gonads and from adult sperm (from Figure 6b and c). The somatic non-germline gonadal cells had both methylated (red) and unmethylated (blue) reads mapping to the somatic ASM site, suggesting 50% methylation, and consistent with the reads seen within haplotype 2 in (a). The sperm cells had only unmethylated (blue) reads mapping to the right hand side of the somatic ASM site, which overlaps the 5' end of the *H19* transcript. In contrast, only methylated (red) reads mapped to the left hand side of the somatic ASM and extended 5' of this region (chr6:282646549-282651558), which we define as the germline ASM. The overlap of the somatic and germline ASM sites contains three CTCF binding sites (chr6:282648824-282648871, chr6:282649557-282649604, chr6:282650923-282650970) – consistent with the *H19* imprint control region identified in wallaby[1]. RNA-sequencing reads from Sandy's liver sample map to the exons (orange box) of the *H19* mRNA. WGBS, whole genome bisulfite sequencing, ASM, allele-specific methylation; CGI, CpG island; SNP, single nucleotide polymorphism.

Supplementary Table 1. Marsupial genome assemblies to date.

Assembly	Assembly Link	Common name	Scientific name	Size (Mb)	Level	Year	WGS accession	Contig N50 (kb)	Scaffold N50 (kb)	BUSCO type?	BUSCO	Submitter	BioProject	Sex
GCA_002099425.1	phaCin_unsw_v4.1 (https://www.ncbi.nlm.nih.gov/datasets/genome/GCF_002099425.1/)	Koala	*Phascolarctos cinereus* (https://www.ncbi.nlm.nih.gov/datasets/taxonomy/38626/)	3,193	Contig	2017	MSTS01	11,588	n/a	mammalia_odb10	C:98.0%[S:97.5%,D:0.6%],F:0.5%,M:1.5%,n:9226	The Earlham Institute	PRJNA359763	F
GCA_019393635.1	mDroGli1.pri (https://www.ncbi.nlm.nih.gov/datasets/genome/GCF_019393635.1/)	Monito del monte	*Dromiciops gliroides* (https://www.ncbi.nlm.nih.gov/datasets/taxonomy/33562/)	3,303	Chromosome	2021	JAHYBR01	38,231	670,776	mammalia_odb10	C:97.3%[S:93.5%,D:3.8%],F:0.6%,M:2.1%,n:9226	Vertebrate Genomes Project	PRJNA728141	F
GCA_011100635.1	mTriVul1.pri (https://www.ncbi.nlm.nih.gov/datasets/genome/GCF_011100635.1/)	Common brushtail	*Trichosurus vulpecula* (https://www.ncbi.nlm.nih.gov/datasets/taxonomy/9337/)	3,359	Chromosome	2020	JAANDE01	4,315	442,560	mammalia_odb10	C:96.8%[S:92.6%,D:4.2%],F:0.7%,M:2.5%,n:9226	Vertebrate Genomes Project	PRJNA562014	M
GCA_900497805.2	bare-nosed wombat genome assembly (https://www.ncbi.nlm.nih.gov/datasets/genome/GCF_900497805.2/)	Common wombat	*Vombatus ursinus* (https://www.ncbi.nlm.nih.gov/datasets/taxonomy/29139/)	3,487	Scaffold	2018	UNPS02	113	28,503	mammalia_odb10	C:96.0%[S:87.3%,D:8.7%],F:1.2%,M:2.7%,n:9226	MRC Institute of Genetics and Molecular Medicine, University of Edinburgh	PRJEB27783	M
GCA_902635505.1	mSarHar1.11 (https://www.ncbi.nlm.nih.gov/datasets/genome/GCF_902635505.1/)	Tasmanian devil	*Sarcophilus harrisii* (https://www.ncbi.nlm.nih.gov/datasets/taxonomy/9305/)	3,087	Chromosome	2019	CACPPN01	62,340	611,347	mammalia_odb10	C:95.5%[S:94.5%,D:1.0%],F:0.9%,M:3.6%,n:9226	SC	PRJEB35073	F
GCA_027887165.1	mMonDom1.pri (https://www.ncbi.nlm.nih.gov/datasets/genome/GCF_027887165.1/)	Gray short-tailed opossum	*Monodelphis domestica* (https://www.ncbi.nlm.nih.gov/datasets/taxonomy/13616/)	3,598	Chromosome	2007	AAFR03	108	59,810	mammalia_odb10	C:91.6%[S:90.3%,D:1.2%],F:2.0%,M:6.4%,n:9226	The Genome Sequencing Platform, The Genome Assembly Team	PRJNA12561	F
GCA_016433145.1	AgileGrace (https://www.ncbi.nlm.nih.gov/datasets/genome/GCF_016433145.1/)	Agile Gracile Mouse Opossum	*Gracilinanus agilis* (https://www.ncbi.nlm.nih.gov/datasets/taxonomy/191870/)	3,701	Chromosome	2021	JADWME01	73	683,522	mammalia_odb10	C:90.4%[S:87.8%,D:2.7%],F:3.2%,M:6.4%,n:9226	Nanjing Normal University	PRJNA565840	M
GCA_016432865.2	AdamAnt (https://www.ncbi.nlm.nih.gov/datasets/genome/GCA_016432865.2/)	Yellow-footed antechinus	*Antechinus flavipes* (https://www.ncbi.nlm.nih.gov/datasets/taxonomy/38775/)	3,193	Chromosome	2021	JADWMD01	51,844	636,718	mammalia_odb10	C:95.4%[S:93.6%,D:1.8%],F:0.7%,M:3.9%,n:9226	Nanjing Normal University	PRJNA565840	M
GCA_016696395.1	USYD_AStu_M (https://www.ncbi.nlm.nih.gov/datasets/genome/GCA_016696395.1/)	Brown antechinus	*Antechinus stuartii* (https://www.ncbi.nlm.nih.gov/datasets/taxonomy/9283/)	3,307	Scaffold	2021	JACYGW01	78	72,733	n/a	n/a	University of Sydney	PRJNA664282	M
GCA_023548195.1	mBetpen1.pri.20210916 (https://www.ncbi.nlm.nih.gov/datasets/genome/GCA_023548195.1/)	Woylie	*Bettongia penicillata ogilbyi* (https://www.ncbi.nlm.nih.gov/datasets/taxonomy/881300/)	3,393	Scaffold	2022	JAJHZJ01	1,995	6,940	n/a	n/a	The University of Sydney	PRJNA763700	F
GCA_020854095.1	UniMelb_DasViv_v1.0 (https://www.ncbi.nlm.nih.gov/datasets/genome/GCA_020854095.1/)	Eastern quoll	*Dasyurus viverrinus* (https://www.ncbi.nlm.nih.gov/datasets/taxonomy/9279/)	3,139	Chromosome	2021	JAJAFV01	13,753	628,487	n/a	n/a	The University of Melbourne	PRJNA758704	F
GCA_011680675.1	LBP_v1 (https://www.ncbi.nlm.nih.gov/datasets/genome/GCA_011680675.1/)	Leadbeater's possum	*Gymnobelideus leadbeateri* (https://www.ncbi.nlm.nih.gov/datasets/taxonomy/38618/)	3,469	Scaffold	2020	WOXC01	501	502	n/a	n/a	Deakin University	PRJNA593327	F
GCA_023553655.1	mMyrfas1.20211206 (https://www.ncbi.nlm.nih.gov/datasets/genome/GCA_023553655.1/)	Numbat	*Myrmecobius fasciatus* (https://www.ncbi.nlm.nih.gov/datasets/taxonomy/55782/)	3,424	Scaffold	2022	JAJPUD01	38	223	n/a	n/a	The University of Sydney	PRJNA786364	F
GCA_000004035.1	Meug_1.1 (https://www.ncbi.nlm.nih.gov/datasets/genome/GCA_000004035.1/)	Tammar wallaby	*Notamacropus eugenii* (https://www.ncbi.nlm.nih.gov/datasets/taxonomy/9315/)	3,075	Scaffold	2009	ABQO01	3	37	n/a	n/a	Tammar Wallaby Genome Sequencing Consortium	PRJNA12587	F

GCA_007646695.3	UniMelb_ThyCyn2.0_hybrid_assembly (https://www.ncbi.nlm.nih.gov/datasets/genome/GCA_007646695.3/)	Tasmanian wolf	*Thylacinus cynocephalus* (https://www.ncbi.nlm.nih.gov/datasets/taxonomy/9275/)	3,377	Chromosome	2022	VAHE03	15	629,086	n/a	n/a	The University of Melbourne	PRJNA354646	F

Supplementary Table 2. Lipocalin gene cluster on Chromosome 3.

Chromosome	Genomic position	Gene	Protein	Protein name
3	173,451,178 - 173,455,153	*LCN9*	XP_036605766.1	Epididymal-specific lipocalin-9
	173,480,469 -173,487,813	*LOC11884295*	XP_036605767.1	Trichosurin-like
	173,578,654 -173,586,395	*LOC118841385*	XP_036604669.1	Trichosurin-like
	173,593,423 - 173,599,643	*LOC118842296*	XP_036605768.1	Major urinary protein-like
	173,670,196 - 173,677,008	*LOC118843263*	XP_036606697.1	Minor allergen Can f 2-like isoform X1
			XP_036606698.1	Major urinary protein 5-like isoform X2
			XP_036606699.1	Major urinary protein 5-like isoform X3
	173,712,002 - 173,722,644	*LOC118842298*	XP_036605769.1	Trichosurin-like
	173,737,858 - 173,743,805	*LOC118844010*	XP_036607713.1	Trichosurin-like
	173,736,995 - 173,852,176	*LOC118844008*	XP_036607712.1	Trichosurin-like
	173,831,353 - 173,837,393	*LOC118844011*	XP_036607714.1	Major urinary protein-like
	173,852,744 - 173,856,236	*LOC118844299*	XP_036605770.1	Minor allergen Can f 2-like
	173,938,493 - 173,944,328	*LOC118841424*	XP_036604713.1	Trichosurin-like
	173,980,033 - 173,988,587	*LOC118841226*	XP_036604510.1	Major urinary protein-like
	174,091,228 - 174,096,488	*LOC118842300*	XP_036605771.1	Major urinary protein-like
	174,110,675 - 174,117,705	*LOC118841314*	XP_036604581.1	Trichosurin

Supplementary Table 3. Primers and PCR conditions used in this study.

Putative Y-chromosome

Gene	Protein title	Scaffold	Primer sequence	PCR product size (bp)	PCR cycling conditions
LOC118833335	Sex-determining region Y protein-like	chrUn_JAANDE010000157v1	TATGCTATGGTCGCGGAGTC	209 bp	98°C, 30 sec; 27x (98°C, 10 sec; 62°C, 20 sec; 72°C, 15 sec); 72°C 2 min
			GAAATTTCTTCCGACGGGGC		
LOC118833342	OTU domain-containing protein 5-like	chrUn_JAANDE010000157v1	GTGGATGTTGCGGTGATAGC	200 bp	
			CACAGGTAAGGCAATGCTGG		
LOC118833329	Synaptonemal complex central element protein 1-like	chrUn_JAANDE010000154v1	GAGGTAAAGCCTTGTTTCAGCC	566 bp	
			AGTTGCTCGCCAAACTCCAT		
LOC118833333	FAM122A-like	chrUn_JAANDE010000154v1	TGTTCCCTCCAACATGATTTTTCC	188 bp	
			GGTGGAAATAATCCCACGGC		
LOC118833356	THO complex subunit 2-like	chrUn_JAANDE010000164v1	TCGGATGTCAAGTTTTCGTCGT	210 bp	
			AAATTCACCGGTTCTCCAGCTGGA		
UBA1	UBA1	chrUn_JAANDE010000164v1	GCTACCCCGTCCCCAAAACCA	185 bp	
			ACAACCTGTGCCAATCCAACCA		

Mitochondrial genome

PCR	Region amplified	Primer sequence	PCR product size (bp)	PCR cycling conditions
1	315 bp - 3566 bp	CCACCGCGGCCATACGATTA	3253 bp	98°C, 30 sec; 27x (98°C, 10 sec; 65°C, 20 sec; 72°C, 2 min); 72°C, 10 min
		CGAAATCGTGGGTATGAGGCTCG		
2	3538 bp - 6937 bp	TGAGTACGAGCCTCATACCCACG	3400 bp	
		TAGGGGGGTTCGATTCCTCCCT		
3	6837 bp - 10314 bp	ATCTTGAATGACTTTATGGT	3478 bp	98°C, 30 sec; 27x (98°C, 10 sec; 65°C, 20 sec; 72°C, 2 min); 72°C, 10 min
		TTAGACTAATTACGCTGATG		
4	10295 bp - 13875 bp	CATCAGCGTAATTAGTCTAA	3581 bp	
		TTATACTTCTGCTATGGCTA		
5	13697 bp - 375 bp	GCCCAACACAATAAACACCAACAGT	3870 bp	98°C, 1 min; 30x (98°C, 20 sec; 65°C, 6 min); 65°C, 7 min
		TGCTTAAACACACTTTACGCCGGT		

Amplification of SNPs in imprinted genes - DNA

Gene	SNP	Genomic coordinate for amplicon	Primer sequence	PCR product size (bp)*	PCR cycling conditions - first round of PCR
H19	chr6:282653107 (+)	chr6:282653001-282653184	ACACTCTTTCCCTACACGACGCTCTTCCGATCTCAGAAAGACCGAAAGAGACCCAGA	251 bp	98°C, 3 min; 27x (98°C, 10 sec; 62°C, 20 sec; 72°C, 15 sec); 72°C, 2 min
			GTGACTGGAGTTCAGACGTGTGCTCTTCCGATCTCTAGTTCGCGCCTCTCCTTCTT		
IGF2	chrUn_JAANDE010000037v1:11329 (-)	chrUn_JAANDE010000037v1:11210-11406	ACACTCTTTCCCTACACGACGCTCTTCCGATCTGAGCTCTGAGGGGAAGGCGG	263 bp	
			GTGACTGGAGTTCAGACGTGTGCTCTTCCGATCTCACCCACACTCACATCCAGGCA		
IGF2R	chr7:94900327 (+)	chr7:94900212-94900445	ACACTCTTTCCCTACACGACGCTCTTCCGATCTCAGATCTTCATCACTGTCGTCATGA	301 bp	

			GTGACTGGAGTTCAGACGTGTGCTCTTCCGATCTGACGAGGTCTTGACCATTCCTGAT		
MLH1	chr9:191436025 (-)	chr9:191435894-191436094	ACACTCTTTCCCTACACGACGCTCTTCCGATCTGCAAATGGCCGCACCTCATCG<br>GTGACTGGAGTTCAGACGTGTGCTCTTCCGATCTCTTCCCCTGTGTGCCTCGCT	268 bp	
GPX7	chr4:27665030 (-)	chr4:27664860-27665060	ACACTCTTTCCCTACACGACGCTCTTCCGATCTGCATTGGCCCCAGTACCACG<br>GTGACTGGAGTTCAGACGTGTGCTCTTCCGATCTAGACCAGCACTACCGAGCCC	268 bp	
MLH1	chr9:191435198 (-)	chr9:191435100-191435323	ACACTCTTTCCCTACACGACGCTCTTCCGATCTCTCTCCCTGCCTGGATCTGGC<br>GTGACTGGAGTTCAGACGTGTGCTCTTCCGATCTTGCAGGGATGAGGCAGAGGG	291 bp	98°C, 3 min; 27x (98°C, 10 sec; 60°C, 20 sec; 72°C, 15 sec); 72°C, 2 min
MLH1	chr9:191445667 (-)	chr9:191445496-191445733	ACACTCTTTCCCTACACGACGCTCTTCCGATCTTTCCTCATCAGGCTGGTCGGC<br>GTGACTGGAGTTCAGACGTGTGCTCTTCCGATCTTCTGCTGCCGGGAGTTGCTG	305 bp	
UBP1	chr9:207243191 (+) and chr9:207243224 (+)	chr9:207243078-207243308	ACACTCTTTCCCTACACGACGCTCTTCCGATCTGTGGGAGCCTCTCACAGCCC<br>GTGACTGGAGTTCAGACGTGTGCTCTTCCGATCTGGCCAGCACCCTTACCTGGT	298 bp	
EPM2AIP1	chr9:191486899 (+)	chr9:191486841-191487058	ACACTCTTTCCCTACACGACGCTCTTCCGATCTACTGGTTCGAAGGCGGGCTG<br>GTGACTGGAGTTCAGACGTGTGCTCTTCCGATCTGCCCACGAACGCCTGGTCAT	285 bp	As above**

Underlined sequence is the 'handle' sequence that is incorporated into amplicon during 1st round of PCR amplification; *includes 67 bp of handle sequences; **add DMSO to PCR reaction (6% final conc.).

Amplification of SNPs in imprinted genes - mRNA

Gene	SNP	Genomic coordinate for amplicon	Primer sequence	PCR product size (bp)$^{\%}$	PCR cycling conditions - first round of PCR
*H19**	chr6:282653107 (+)	chr6:282653001-282653184	ACACTCTTTCCCTACACGACGCTCTTCCGATCTCAGAAAGACCGAAAGAGACCCAGA<br>GTGACTGGAGTTCAGACGTGTGCTCTTCCGATCTCTAGTTCGCGCCTCTCCTTCTT	251 bp	98°C, 3 min; 27x (98°C, 10 sec; 62°C 20 sec, 72°C, 15 sec); 72°C 2 min
*IGF2R**	chr7:94900327 (+)	chr7:94900212-94900445	ACACTCTTTCCCTACACGACGCTCTTCCGATCTCAGATCTTCATCACTGTCGTCATGA<br>GTGACTGGAGTTCAGACGTGTGCTCTTCCGATCTGACGAGGTCTTGACCATTCCTGAT	301 bp	
MLH1	chr9:191436025 (-)	chr9:191435175-191436066 (genomic)<br>*MLH1*:1699-1934 (CDS)	ACACTCTTTCCCTACACGACGCTCTTCCGATCTGCACCCCTATACGACCTGGCC<br>GTGACTGGAGTTCAGACGTGTGCTCTTCCGATCTAGCACGAACATGGGAAGCCC	303 bp	
GPX7	chr4:27665030 (-)	chr4:27664950-27707162 (genomic)<br>*GPX78*:111-303 (CDS)	ACACTCTTTCCCTACACGACGCTCTTCCGATCTTTGGTGTCTGGCTCCTGCTGG<br>GTGACTGGAGTTCAGACGTGTGCTCTTCCGATCTTCAGGGGCAAGTGGGTGTCC	260 bp	
MLH1$^{\#}$	chr9:191435198 (-)	chr9:191435175-191436066 (genomic)<br>*MLH1*:1699-1934 (CDS)	ACACTCTTTCCCTACACGACGCTCTTCCGATCTGCACCCCTATACGACCTGGCC<br>GTGACTGGAGTTCAGACGTGTGCTCTTCCGATCTAGCACGAACATGGGAAGCCC	291 bp	
MLH1	chr9:191445667 (-)	chr9:191445496-191445733	ACACTCTTTCCCTACACGACGCTCTTCCGATCTTTCCTCATCAGGCTGGTCGGC<br>GTGACTGGAGTTCAGACGTGTGCTCTTCCGATCTTCTGCTGCCGGGAGTTGCTG	305 bp	
UBP1	chr9:207243191 (+) and chr9:207243224 (+)	chr9:207237856-207243270 (genomic)<br>*UBP1*:1324-1510 (CDS)	ACACTCTTTCCCTACACGACGCTCTTCCGATCTAGGGGTCCGCAGCTGGAAAG<br>GTGACTGGAGTTCAGACGTGTGCTCTTCCGATCTTCCCGGTGGGACCCTGTCT	254 bp	
*EPM2AIP1**	chr9:191486899 (+)	chr9:191486841-191487058	ACACTCTTTCCCTACACGACGCTCTTCCGATCTACTGGTTCGAAGGCGGGCTG<br>GTGACTGGAGTTCAGACGTGTGCTCTTCCGATCTGCCCACGAACGCCTGGTCAT	285 bp	98°C, 3 min; 27x (98°C, 10 sec; 60°C, 20 sec; 72°C, 15 sec); 72°C 2 min

Underlined sequence is the 'handle' sequence that is incorporated into amplicon during 1st round of PCR amplification; *same primers as for DNA amplification above; #same primers as for SNP chr9:191436025 (-); $^{\%}$includes 67 bp of handle sequences

Indexed Truseq-type oligos for Illumina seqeuncing

Primer ID	Linker (handle) sequence	Index and Illumina adpter sequence	Full primer sequence
Illumina P5 adapter with TruSeq index	ACACTCTTTCCCTACACGACGCTCTTCCGATCT	AATGATACGGCGACCACCGAGATCTACACNNNNNNNN	AATGATACGGCGACCACCGAGATCTACACNNNNNNNNACACTCTTTCCCTACACGACGCTCTTCCGATCT
Illumina P7 adapter with TruSeq index	GTGACTGGAGTTCAGACGTGTGCTCTTCCGATCT	CAAGCAGAAGACGGCATACGAGATNNNNNNNN	CAAGCAGAAGACGGCATACGAGATNNNNNNNNGTGACTGGAGTTCAGACGTGTGCTCTTCCGATCT

NNNNNNNN represents the unique 8 bp index.

Supplementary Table 4. Statistics and coverage of nanopore data.

Run	Library preparation method	Mean read length (bp)	Mean read quality	Number of reads	Read length N50 (bp)	Longest read (bp)	Total bases
1	Rapid Sequencing Kit	5,047.5	14.5	515,843	8,898	147,804	2,603,711,950
2	Ligation Sequencing Kit	8,000.8	14	447,900	14,166	970,296	3,587,119,132
3	Ligation Sequencing Kit	7,958.3	14	1,897,244	15,097	188,418	15,098,845,291
4	Ligation Sequencing Kit	8,351.1	13.5	1,423,400	15,660	189,901	11,887,018,680
5	Ligation Sequencing Kit	8,117.4	14	1,730,032	15,396	207,614	14,043,331,027
						**Grand total bases**	47,220,026,080
						**Genome size (bases)**	3,359,347,707
						**Coverage**	14.05X

Supplementary Table 5. Genomic position of SNPs used to determine mono-allelic expression.

Chromosome	mRNA	Genomic position of SNP	SNP present in Sandy?	How was SNP identified?
chr7	*IGF2R*	94900327	Yes	Nanopore data - SNP detection in Sandy's genome
chr6	*H19*	282653107	No	Reese, 2021[2]
chrUn_JAANDE010000037v1	*IGF2*	11329	No	RNA-sequencing data – variant identified in population
chr9	*MLH1*	191435198	Yes	Nanopore data - SNP detection in Sandy's genome
chr9	*MLH1*	191436025	No	RNA-sequencing data – variant identified in population
chr9	*MLH1*	191445667	No	RNA-sequencing data – variant identified in population
chr9	*EPM2AIP1*	191486899	Yes	Nanopore data - SNP detection in Sandy's genome
chr9	*UBP1*	207243191	Yes	Nanopore data - SNP detection in Sandy's genome
chr9	*UBP1*	207243224	No	RNA-sequencing data – variant identified in population
chr4	*GPX7*	27665030	Yes	Nanopore data - SNP detection in Sandy's genome

References

1. Smits, G., Mungall, A., Griffiths-Jones, S. *et al*. Conservation of the *H19* noncoding RNA and *H19-IGF2* imprinting mechanism in therians. *Nat. Genet.* **40**, 971–976 (2008).
2. Reese, F. (2021) Investigating Genomic Imprinting in the Brushtail Possum. (University of Otago, 2021).

Uncropped gel photos used to generate Fig. S1b:

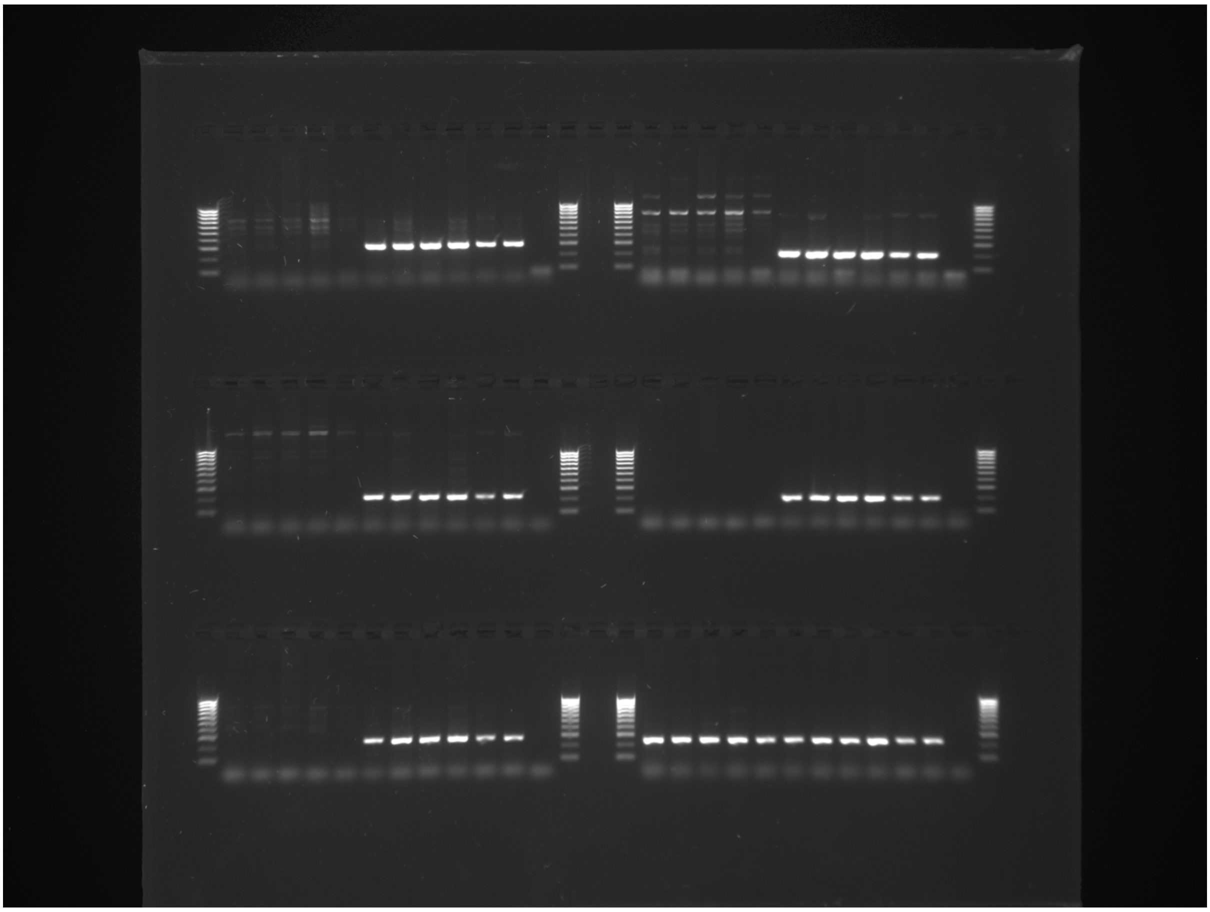

Gel file name: 4-3-21

LOC118833333 (*FAM122A-like*) – middle, right

LOC118853838 (*H19*) – bottom, right

Uncropped gel photos used to generate Fig. S1b:

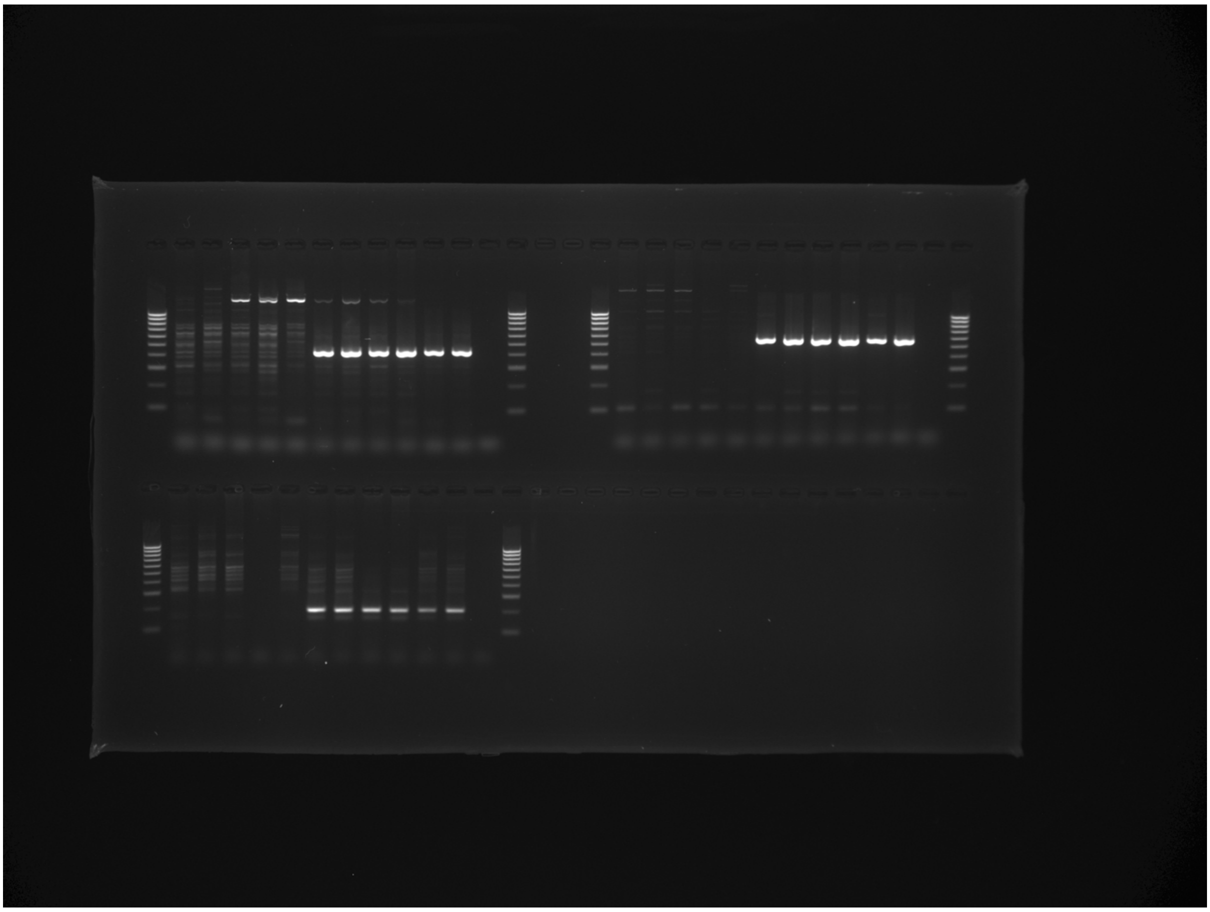

Gel file name: 18-5-21

LOC118833329 (*Synaptonemal complex central element protein 1-like*) – top, right

Uncropped gel photos used to generate Fig. S1b:

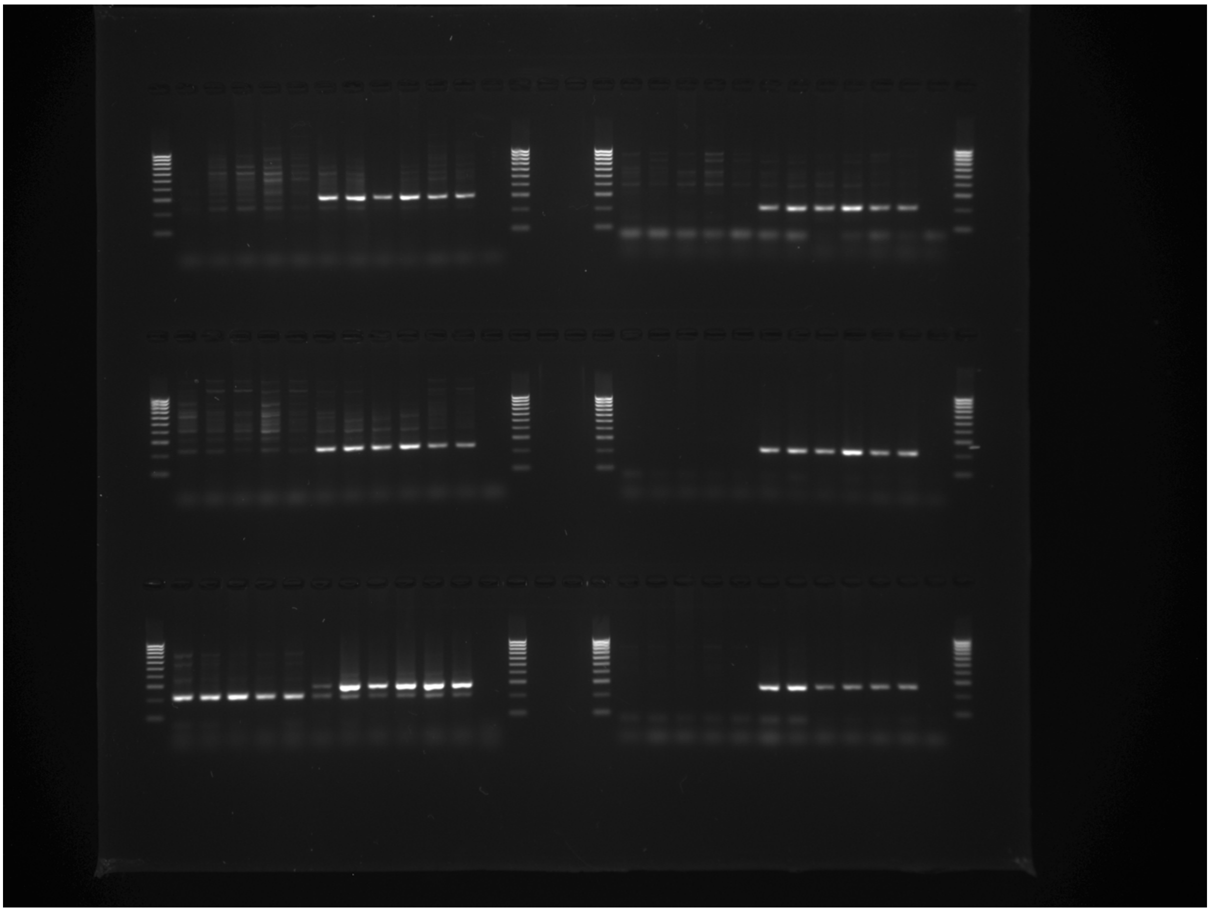

Gel file name: 2-6-21_2nd

LOC118833342 (*OTU domain-containing protein 5-like*) – top, right

LOC118833335 (*Sex-determining region Y protein-like*) – middle, right

Uncropped gel photos used to generate Fig. S1b:

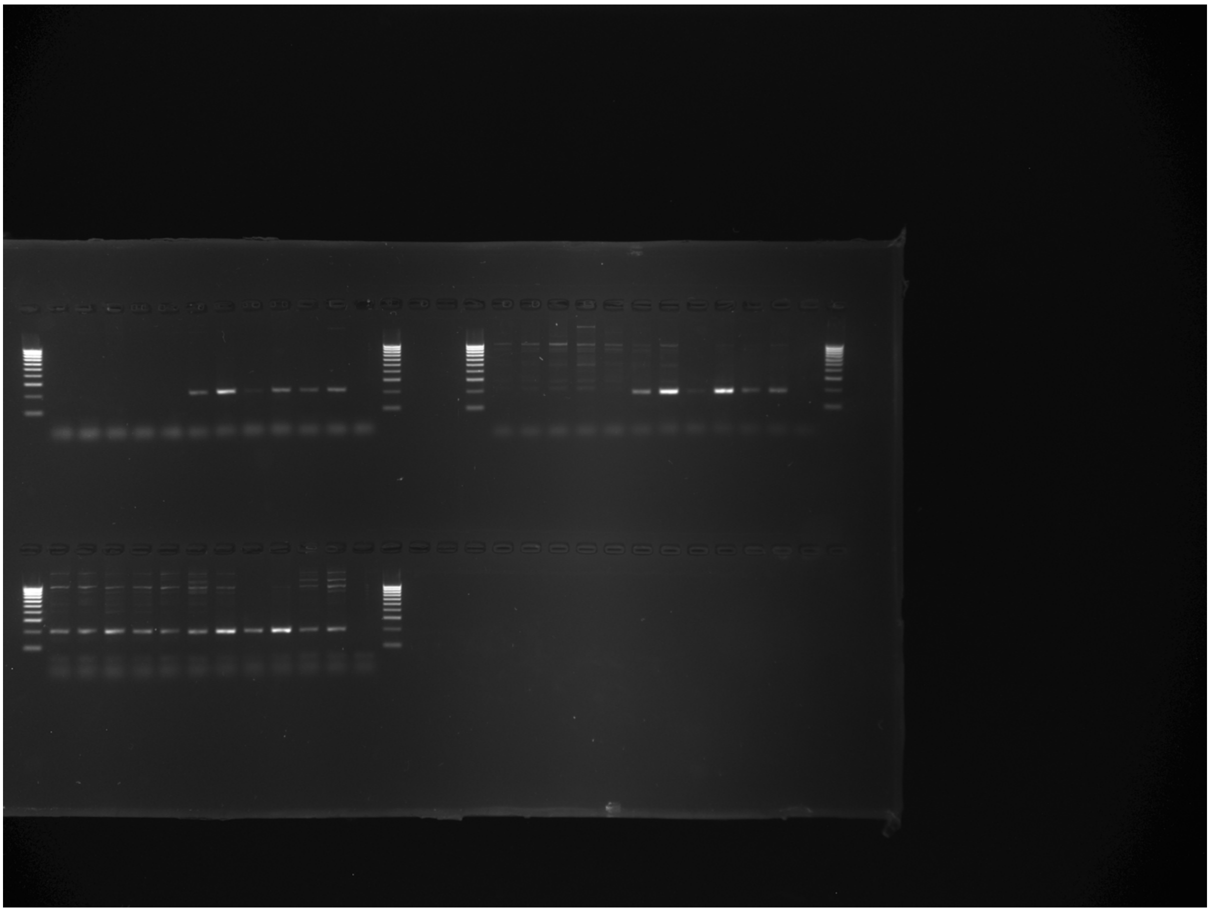

Gel file name: 2-11-22

LOC118833356 (*THO complex subunit 2-like*) – top, left

UBA1 – top, right
