# Supplementary material for: The admixed brushtail possum genome reveals invasion history in New Zealand and novel imprinted genes
Source: Nat Commun. 2023 Oct 17;14:6364. doi: 10.1038/s41467-023-41784-8 (PMC10582058; doi:10.1038/s41467-023-41784-8)
Supplement: Supplementary file 3 — Reporting Summary [file 41467_2023_41784_MOESM3_ESM.pdf]

nature portfolio

Corresponding author(s): Timothy Hore
Last updated by author(s): 18/8/2023

Reporting Summary

Nature Portfolio wishes to improve the reproducibility of the work that we publish. This form provides structure for consistency and transparency in reporting. For further information on Nature Portfolio policies, see our Editorial Policies and the Editorial Policy Checklist.

Statistics

For all statistical analyses, confirm that the following items are present in the figure legend, table legend, main text, or Methods section.

n/a	Confirmed	
☐	☒	The exact sample size (*n*) for each experimental group/condition, given as a discrete number and unit of measurement
☐	☒	A statement on whether measurements were taken from distinct samples or whether the same sample was measured repeatedly
☐	☒	The statistical test(s) used AND whether they are one- or two-sided<br>*Only common tests should be described solely by name; describe more complex techniques in the Methods section.*
☐	☒	A description of all covariates tested
☐	☒	A description of any assumptions or corrections, such as tests of normality and adjustment for multiple comparisons
☐	☒	A full description of the statistical parameters including central tendency (e.g. means) or other basic estimates (e.g. regression coefficient) AND variation (e.g. standard deviation) or associated estimates of uncertainty (e.g. confidence intervals)
☐	☒	For null hypothesis testing, the test statistic (e.g. *F*, *t*, *r*) with confidence intervals, effect sizes, degrees of freedom and *P* value noted<br>*Give P values as exact values whenever suitable.*
☐	☒	For Bayesian analysis, information on the choice of priors and Markov chain Monte Carlo settings
☒	☐	For hierarchical and complex designs, identification of the appropriate level for tests and full reporting of outcomes
☐	☒	Estimates of effect sizes (e.g. Cohen's *d*, Pearson's *r*), indicating how they were calculated

Our web collection on statistics for biologists contains articles on many of the points above.

Software and code

Policy information about availability of computer code

Data collection	No software for data collection was used.
Data analysis	A full description of software used for data analysis, along with parameters used, is listed in the methods.<br><br>Software for whole genome sequencing analysis: Trim Galore! (v0.6.7) to remove adapters and low-quality base calls (Phred score < 20); BWA-MEM algorithm (v0.7.17) for mapping; SAMtools (v1.15.1) for SAM to BAM conversion; R package GenomicRanges (v1.38.0) to determine sequencing depth (non-overlapping 1 kilobase windows were created for all chromosomes and contigs); BEDtools (v2.30.0) to determine the number of reads mapping to each window (coverage command).<br><br>Software for RNA-sequencing and coverage analysis: Trim Galore! (v0.6.7) to remove adapters and low-quality base calls (Phred score < 20); HISAT2 (v2.2.1) for mapping; SeqMonk (v1.48.0) for data visualisation and RNAseq quantitation pipeline.<br><br>Software for SNP calling using short reads: GATK's Best Practices (v4.2.6.1), Duplicate reads (were marked using the MarkDuplicates GATK module and read group tags were added using the addreplacerg function from SAMtools (v1.15.1). SNPs were called into gVCF files for each sample using the HaplotypeCaller GATK module. Individual gVCFs were combined using the CombineGVCFs GATK module and genotypes of the samples were obtained with the GenotypeGVCFs GATK module. High-quality SNPs were filtered with VCFtools (v0.1.15) using the following parameters: --minQ 30 --min-meanDP 10 --max-missing 0.95.<br><br>Software for mitochondrial genome assembly: Trim Galore! (v0.6.7) to remove adapters and low-quality base calls (Phred score < 20, length <35). BWA-MEM algorithm (v0.7.17) for mapping; SAMtools (v1.15.1) for SAM to BAM conversion; FASTQ consensus sequences were

March 2021

generated using the mpileup command from BCFtools (v1.15.1).

Software for mitochondrial genome phylogenetic and spatial analyses: Geneious alignment sing Geneious v 2022.2.2; Maximum likelihood (ML) trees were constructed via RAxML v 8.2.12 (100 bootstrap replicates) and two separate runs were used to confirm convergence, utilising the GTRCAT model of substitution; verified results using BEAST v 2.6.6; Tracer v 1.7.2 was used to check convergence of parameters and topologies of the consensus trees; TreeAnnotator for tree construction (maximum clade credibility tree, common ancestor heights); R v 4.2.1 and the tidyverse v 1.1.2, ggmap v 3.0.0, and ggrepel v 0.9.1 packages to examine the spatial distribution of mitochondrial haplotypes in the greater Dunedin region.

Software for nuclear population genomics: R utilising the inbreedR, vcfR, reshape, and future.apply packages for standardized multilocus heterozygosity (sMLH) calculations; tidyverse and rstatix v 0.7.0 packages for sMLH comparisons; PLINK v1.90b6.21 to remove closely related individuals (three consecutive rounds of sample filterin) with threshold of PI_HAT scores >0.25, and pca command; ADMIXTURE v1.3.0 to determine genetic clusters; ggplot v3.3.6 package in the R language plotting results.

Software for identification of allele-specific methylation by nanopore sequencing data: Guppy v6.2.1 (uppy_basecaller -i FAST5 -c dna_r9.4.1_450bps_sup.cfg -x “cuda:0” -s FASTQ) for basecalking; trimmed using Porechop (v0.2.4), quality filtered using Filtlong (v0.2.0), Minimap2 (v2.24, “map-ont” parameter) for mapping; SAMtools (v1.15.1) bedcov using to estimate coverage; Nanopolish (v0.13.3) call-methylation module for CpG methylation calls; Clair (v2.1.1) for methylation phasing (SNP detection were set at a threshold of 0.2 and the 122HD34 was selected as the precomputed model); SNVoter (v.1.0) was used to improve SNP detection from low-coverage regions; SNPs were phased using WhatsHap (v1.1) with default parameters and the --ignore-read-groups; NanoMethPhase (v1.0) to obtain phased CpG methylation and mock bis-sequencing BAM files; IGV (v2.12.3) to visualise BAM files; DMLtest function of the DSS package (v2.34.0) to determine differentially methylated CpG sites; SeqMonk (v1.48.1) for annotation of differentially methylated CpG sites.

Software for mono-allelic analysis of known and candidate genes: SAMtools (v1.9) mpileup (max depth 0) for read depth.

Software for identification of genome wide mono-allelic SNP expression: CollectAllelicCounts function from the GATK toolkit (v4.2.6.1) to quantify the number of reads with the reference and alternative alleles; SNPs were annotated using SeqMonk (v1.48.0); IGV (v2.12.3) was then used to visualise the SNPs.

Software for DNA methylation analysis: Trim Galore! (v0.6.7) to remove adapters and low-quality base calls (Phred score < 20) and 10bp from the 5’ end of all reads; Bismark (v0.19.0) with the -pbat option was used for mapping (low coverage) or Bismark v0.22.3 for mapping and deduplication; SeqMonk (v1.48.0) for visualisation of BAM files, feature probe generation and Difference Quantitation method with minimum read count=1.

Software for amplicon sequencing analysis: Geneious v2022.2.2 for trimming (BBDuk plugin) and determining nucleotide sequence at SNP (Find Variations/SNPs – minimum variant frequency of 0.2).

The source code of the analysis is publicly available on GitHub at https://github.com/TimHore-Otago/Bond_NZ_brushtail_possum and https://github.com/laninsky/possums/ (geospatial, mitogenomes, and sMLH folders).

For manuscripts utilizing custom algorithms or software that are central to the research but not yet described in published literature, software must be made available to editors and reviewers. We strongly encourage code deposition in a community repository (e.g. GitHub). See the Nature Portfolio guidelines for submitting code & software for further information.

Data

Policy information about availability of data

All manuscripts must include a data availability statement. This statement should provide the following information, where applicable:

- Accession codes, unique identifiers, or web links for publicly available datasets
- A description of any restrictions on data availability
- For clinical datasets or third party data, please ensure that the statement adheres to our policy

The genome assembly data generated in this study has been deposited in the NCBI BioProject database under accession: PRJNA562248 [https://www.ncbi.nlm.nih.gov/bioproject/?term=PRJNA562248]. The RNA-sequencing and bisulfite sequencing data generated in this study has been deposited to the NCBI Gene Expression Omnibus (GEO) database under accession: GSE218695 (NCBI BioProject: PRJNA904814; [https://www.ncbi.nlm.nih.gov/bioproject/?term=PRJNA904814]) and GSE218734 (NCBI BioProject: PRJNA905369; [https://www.ncbi.nlm.nih.gov/bioproject/?term=PRJNA905369]), respectively. The raw data for the mitochondrial DNA libraries and nanopore libraries generated in this study has been deposited in the NCBI Short Read Archive under accession: PRJNA904809 [https://www.ncbi.nlm.nih.gov/bioproject/?term=PRJNA904809]. The possum reference genome and annotation used in this study are available in the NCBI database under accession: GCA_011100635.1 [https://www.ncbi.nlm.nih.gov/datasets/genome/GCA_011100635.1/]. The possum reference mitochondrial genome used in this study are available in the NCBI database under accession code: NC_003039.1 [https://www.ncbi.nlm.nih.gov/nuccore/NC_003039.1]. Various reference samples used in this study are in the NCBI database under accession: PRJNA623153 [https://www.ncbi.nlm.nih.gov/bioproject/?term=PRJNA623153], PRJNA323970 [https://www.ncbi.nlm.nih.gov/bioproject/?term=PRJNA323970], PRJNA525264 [https://www.ncbi.nlm.nih.gov/bioproject/?term=PRJNA525264] and PRJNA587034 [https://www.ncbi.nlm.nih.gov/bioproject/?term=PRJNA587034] - see Supplementary Data 5 for further details. Consensus sequences of assembled mitochondrial genomes used in this study are given in Supplementary Data 7. The raw amplicon sequencing data generated in this study has been deposited to Github (https://github.com/TimHore-Otago/Bond_NZ_brushtail_possum). Source data are provided as a Source Data file.

Human research participants

Policy information about studies involving human research participants and Sex and Gender in Research.

Reporting on sex and gender	N/A
Population characteristics	N/A
Recruitment	N/A
Ethics oversight	N/A

Note that full information on the approval of the study protocol must also be provided in the manuscript.

Field-specific reporting

Please select the one below that is the best fit for your research. If you are not sure, read the appropriate sections before making your selection.

☒ Life sciences ☐ Behavioural & social sciences ☐ Ecological, evolutionary & environmental sciences

For a reference copy of the document with all sections, see nature.com/documents/nr-reporting-summary-flat.pdf

Life sciences study design

All studies must disclose on these points even when the disclosure is negative.

Sample size	Sample size involved field collection of >100 possum samples. Samples were chosen to represent developmental stages from immediately post-birth until weaning, juveniles and into adulthood.<br>For RNA-sequencing analysis: samples were assigned to a group based on age (pouch young (0-120 days post birth); juvenile pouch young (>120 days post birth, with mother); or adult (not present with mother)). The sample sizes of the three groups (33 pouch young, 7 juvenile pouch young and 44 adults) provide sufficient numbers of animals per group for extrapolation of data.<br>For methylation/reprogramming analysis: we used all the pouch young samples we had access to. Based on their age, these samples fell into three groups: 'pre' methylation loss (<10dpp, n=1), 'demethylated' (13-16 dpp, n=3) and 'remethylated' (>16dpp, n=12). As the sample size of the 'pre' methylation group is insufficient to draw conclusions from the data, we have included it in the raw data figure (Fig 6b) but excluded it from statistical analysis and our interpretations. The sample sizes of the other two groups provide sufficient replication for statistical analysis (ttest) and extrapolation of data.
Data exclusions	Samples were removed from the analysis on account of technical issues related to RNA-sequencing library construction or sequencing only (e.g low read number, poor library construction etc). Only small numbers were affected by this (~2 out of 116 for liver RNA-sequencing) and outlined in the manuscript. See above for data exclusions for methylation/reprogramming analyses.
Replication	We had 3 different sample sites, as well as possums reared in captivity, and collected over a 3 year period. For RNA-sequencing analysis we had minor changes in the library construction method - there was no clustering based on any of these factors - rather, we just found clustering by developmental stage.
Randomization	Possums were assigned ages using the nomogram of Lyne and Verhagen.<br>For RNA-sequencing analysis: individual pouch young samples were allocated to groups based on age (identified by measuring head length and comparing it against a published nomogram).<br>For methylation/reprogramming analysis: individual pouch young samples were allocated to groups based on age (identified by measuring head length and comparing it against a published nomogram) and by global % CpG methylation of germ cells.
Blinding	Collection of tissue was performed by different individuals to those doing library construction, sequencing and analysis.

Reporting for specific materials, systems and methods

We require information from authors about some types of materials, experimental systems and methods used in many studies. Here, indicate whether each material, system or method listed is relevant to your study. If you are not sure if a list item applies to your research, read the appropriate section before selecting a response.

Materials & experimental systems

n/a	Involved in the study	
	x	Antibodies
x		Eukaryotic cell lines
x		Palaeontology and archaeology
	x	Animals and other organisms
x		Clinical data
x		Dual use research of concern

Methods

n/a	Involved in the study	
x		ChIP-seq
	x	Flow cytometry
x		MRI-based neuroimaging

Antibodies

Antibodies used	We used a conjugated primary antibody for labelling germ cells. The details are: SSEA1 monoclonal antibody (Invitrogen, MC-480) conjugated to DyLight 488 (1:300 in 5 % FBS (in PBS); Invitrogen, MA1-022-D488).
Validation	Very few antibodies are validated for use in marsupial tissue by manufacturers (e.g. the antibody we used is validated by Invitrogen for use with human and mouse tissue). However, we have validated this antibody for germ cell specificity in brushtail possum in a previous manuscript (Laird MK, Hore TA. 2021. Primordial germ cell expression of SSEA1 and DDX4 (VASA) in female Trichosurus vulpecula (Marsupialia) reveals conserved and unique molecular patterns during marsupial germ cell development. Reprod Fertil Devel DOI: 10.1071/RD20203).

Animals and other research organisms

Policy information about studies involving animals; ARRIVE guidelines recommended for reporting animal research, and Sex and Gender in Research

Laboratory animals	No laboratory animals were used in this study.
Wild animals	Samples from adult male and female brushtail possums (Trichosurus vulpecula) were collected in one of two ways. Tissue was collected either opportunistically from animals trapped and culled by local trapping operators, or from animals we trapped ourselves using Grieve wire cage traps. These animals were killed at the site of trapping using a pellet gun. Euthanasia is necessary to obtain the samples we required (e.g. liver) and trapped possums cannot be legally released as they are a pest species in NZ. All animal usage is approved by our institutional Animal Ethics Committee.<br><br>For methylation analysis: We collected pouch young (7-106 dpp) for this experiment in two ways. Some pouch young were collected opportunistically from local trapping operators from females that had been culled but had a young in their pouch. We obtained other pouch young via breeding. For this, we trapped adult male and female brushtail possums (Trichosurus vulpecula) locally (near Dunedin, NZ) using Grieve wire cage traps. We transported these possums by vehicle a short distance (< 30 min) to a holding facility (see details below) where they were used to establish a breeding colony. Pouch young were culled (using sodium pentobarbital injection) to collect gonads for methylation analyses. All animal usage is approved by our institutional Animal Ethics Committee.
Reporting on sex	Sex was recorded and commented on when relevant for interpretation of our findings (e.g. reporgramming of male primordial germ cells, male-enhanced expression of vulpeculin)
Field-collected samples	Possums in our captive breeding colony are housed in a purpose built facility consisting of large pens (3x5x3m WxDxH) that are naturally ventilated/semi-open. Housing is under natural photoperiod and temperature. Possums are housed in small groups (n=3-5) and fed fresh fruit and sheep pellets ad libitum, as per a published protocol (McLeod BJ, Thompson EG, Crawford JL, Shackell GH. 1997. Successful group housing of wild-caught brushtail possums (Trichosurus vulpecula). Animal Welfare 6:67-76). Pouch young born in our colony are euthanised via injection of sodium pentobarbital and tissue harvested for various experiments. Adults are retained in the colony until they fail to breed or the experiment ends, at which point they are euthanised. All animal usage is approved by our institutional Animal Ethics Committee.
Ethics oversight	Tissue samples were sourced from freshly deceased possums killed as part of a pest-control programme, and therefore not requiring animal ethics oversight according to guidelines issued by the NAEAC, Occasional Paper No 2, 2009 (ISBN 978-0-478-33858-4). Additional samples were collected from unused tissue belonging to captive possums euthanised from another study. Capture, husbandry and manipulation in that study was approved by the Otago Animal Ethics Committee (AUP-19-75, AUP-20-10). Further samples for mitogenome analysis were sourced from a prior study.

Note that full information on the approval of the study protocol must also be provided in the manuscript.

Flow Cytometry

Plots

Confirm that:

☒ The axis labels state the marker and fluorochrome used (e.g. CD4-FITC).

☒ The axis scales are clearly visible. Include numbers along axes only for bottom left plot of group (a 'group' is an analysis of identical markers).

☒ All plots are contour plots with outliers or pseudocolor plots.

☒ A numerical value for number of cells or percentage (with statistics) is provided.

Methodology

Sample preparation	Gonads were collected from male pouch young (n=17; Supplementary Data 5) and germ cells were isolated from the gonads as previously described. Briefly, gonads were torn using needles in a droplet of TrypLIExpress (Gibco, 12605010) and incubated at 37°C for 20 min. Tissue clumps were triturated manually by gentle strokes with a P1000 pipette tip then with a P200 pipette tip. TrypLIExpress was diluted with 5 % FBS (in PBS), filtered through a 40 µm cell strainer and washed twice in PBS (centrifugation 240 × g1,500 rpm for 2 min). The resulting pellet was resuspended in Zombie Near-Infra Red Viability Dye (BioLegend, cat. no. 423105) and incubated at room temperature for 20 min. The lysate was washed in 5 % FBS (in PBS), then resuspended in SSEA1 monoclonal antibody (Invitrogen, MC-480) conjugated to DyLight 488 (1:300 in 5 % FBS (in PBS); Invitrogen, MA1-022-D488) on ice for 30 min. The lysate was then washed, resuspended in sort buffer (5% FBS, 0.5 mM EDTA in PBS) and kept on ice prior to cell sorting.
Instrument	BD Biosciences FACSAria Fusion which detects GFP using a 488 argon laser. Further details on the machine can be found here https://www.otago.ac.nz/omni/flow-cytometry/otago830927.html
Software	BD FACS Diva
Cell population abundance	The number of GFP-positive sorted cells varied between 3 and 516 per sample (see Supplementary Data 5). GFP-negative cells (somatic cells) were also sorted from each individual as negative controls (see Supplementary Data 5).
Gating strategy	A gonadal cell profile was produced using forward scatter (FSC) and side scatter (SSC) and was gated to exclude doublets and dead cells. Germ cells were identified as a small sub-population that were very bright (GFP >104) with higher SSC (see Fig. 6a and Supplementary Data 6; approx. 0.1 % of total cells). This population was gated conservatively, sorted into 0.2 % Triton-X and stored at -80 °C.

☒ Tick this box to confirm that a figure exemplifying the gating strategy is provided in the Supplementary Information.
