# Supplementary material for: The admixed brushtail possum genome reveals invasion history in New Zealand and novel imprinted genes
Source: Nat Commun. 2023 Oct 17;14:6364. doi: 10.1038/s41467-023-41784-8 (PMC10582058; doi:10.1038/s41467-023-41784-8)
Supplement: Supplementary file 4 — Description of Additional Supplementary Files [file 41467_2023_41784_MOESM4_ESM.pdf]

Description of Additional Supplementary Files

Supplementary Data 1
Description: Expression of possum globin genes during liver development. RPKM ($\log_2$) determined from RNA-sequencing data.

Supplementary Data 2
Description: Expression levels of genes correlated with weaning.

- Tab 1. Expression GAL+CYP paralogues
  Expression levels of galactose metabolism and cytochrome P450 paralogues associated with weaning - these paralogues were used to generate the average 'GAL' and 'CYP' expressions levels presented in Fig. 2a
- Tab 2. Expression all genes
  Expression levels of all genes - sorted based on their correlation to the GAL/GYP ratio.

Supplementary Data 3
Description: Allele specific methylation sites and associated genes.

- Tab 1. All ASM sites
  Genomic coordinates and features for all allele specific methylation sites identified via nanopore sequencing.
- Tab 2. ASMS associated with mono-allelic expression (MAE)
  Genes with SNPs displaying mono-allelic expression that are associated with ASM sites that display germline reprogramming indicative of imprinting.

Supplementary Data 4
Description: Monoallelic expression of imprinted genes.

- Tab 1. Sandy
  Monoallelic expression of single nucleotide polymorphisms within H19, IGF2, IGF2R, UBP1, GPX7, EPM2AIP1 and MLH1 in RNA-sequencing data from Sandy's tissues.
- Tab 2. H19 chr6:282653107
  Monoallelic expression of single nucleotide polymorphism (chr6:282653107) within H19 in liver RNA-sequencing data, including Hardy-Weinberg equilibrium calculations.
- Tab 3. IGF2 chrUn_JAANDE010000037v1:11329
  Monoallelic expression of single nucleotide polymorphism (chrUn_JAANDE010000037v1:11329) within IGF2 in liver RNA-sequencing data, including Hardy-Weinberg equilibrium calculations.
- Tab 4. IGF2R chr7:94900327
  Monoallelic expression of single nucleotide polymorphism (chr7:94900327) within IGF2R in liver RNA-sequencing data, including Hardy-Weinberg equilibrium calculations.
- Tab 5. UBP1 chr9:207243191
  Monoallelic expression of single nucleotide polymorphism (chr9:207243191) within UBP1 in liver RNA-sequencing data, including Hardy-Weinberg equilibrium calculations.

- Tab 6. UBP1 chr9:207243224
  Monoallelic expression of single nucleotide polymorphism (chr9:207243224) within UBP1 in liver RNA-sequencing data, including Hardy-Weinberg equilibrium calculations.
- Tab 7. GPX7 chr4:27665030
  Monoallelic expression of single nucleotide polymorphism (chr4:27665030) within GPX7 in liver RNA-sequencing data, including Hardy-Weinberg equilibrium calculations.
- Tab 8. EPM2AIP1 chr9:191486899
  Monoallelic expression of single nucleotide polymorphism (chr9:191486899) within EPM2AIP1 in liver RNA-sequencing data, including Hardy-Weinberg equilibrium calculations.
- Tab 9. MLH1 chr9:191435198
  Monoallelic expression of single nucleotide polymorphism (chr9:191435198) within MLH1 in liver RNA-sequencing data, including Hardy-Weinberg equilibrium calculations.
- Tab 10. MLH1 chr9:191436025
  Monoallelic expression of single nucleotide polymorphism (chr9:191436025) within MLH1 in liver RNA-sequencing data, including Hardy-Weinberg equilibrium calculations.
- Tab 11. MLH1 chr9:191445667
  Monoallelic expression of single nucleotide polymorphism (chr9:191445667) within MLH1 in liver RNA-sequencing data, including Hardy-Weinberg equilibrium calculations.
- Tab 12. RT-PCR analysis
  Monoallelic expression of various SNPs via RT-PCR analysis.
- Tab 13. Total number of duos
  Mother/pouch young duos to determine parent of origin expression.

Supplementary Data 5
Description: Metadata for all samples.

- Tab 1. RNAseq metadata
  Metadata for RNAseq libraries
- Tab 2. WGBS metadata
  Metadata for WGBS libraries, including global methylation levels.
- Tab 3. MitoDNA metadata
  Metadata for mitoDNA libraries.
- Tab 4. Reference samples metadata
  Metadata for Reference samples used in this study.

Supplementary Data 6
Description: Flow cytometry plots of samples used for WGBS.

Supplementary Data 7

Description: Mitochondrial genome assemblies.
Consensus sequences for all mitochondrial genomes analysed (.fasta format).
