# Supplementary material for: The admixed brushtail possum genome reveals invasion history in New Zealand and novel imprinted genes
Source: Nat Commun. 2023 Oct 17;14:6364. doi: 10.1038/s41467-023-41784-8 (PMC10582058; doi:10.1038/s41467-023-41784-8)
Supplement: Supplementary file 10 — Supplementary Data 6 [file 41467_2023_41784_MOESM10_ESM.pdf]

Supplementary Data 6. Flow cytometry plots of samples used for WGBS.

The admixed brushtail possum genome reveals invasion history in New Zealand and novel imprinted genes

Bond et al.

This pdf file contains flow cytometry plots for the following samples:

Page	Sample name	Age (dpp)
2	140421_PY01	7
3	110822_PY01	12
4	090421_PY02	13
5	140421_PY02	16
6	250822_PY02	26
7	081021_PY01	27
8	140421_PY03	38
9	290520_PY01	45
10	140722_PY03	50
11	210619_PY02	60
12	270520_PY01	63
13	280520_PY01	69
14	040822_PY02	76
15	300719_PY02	91
16	140720_PY01	102
17	300719_PY04	106

BD FACSDiva 8.0.2

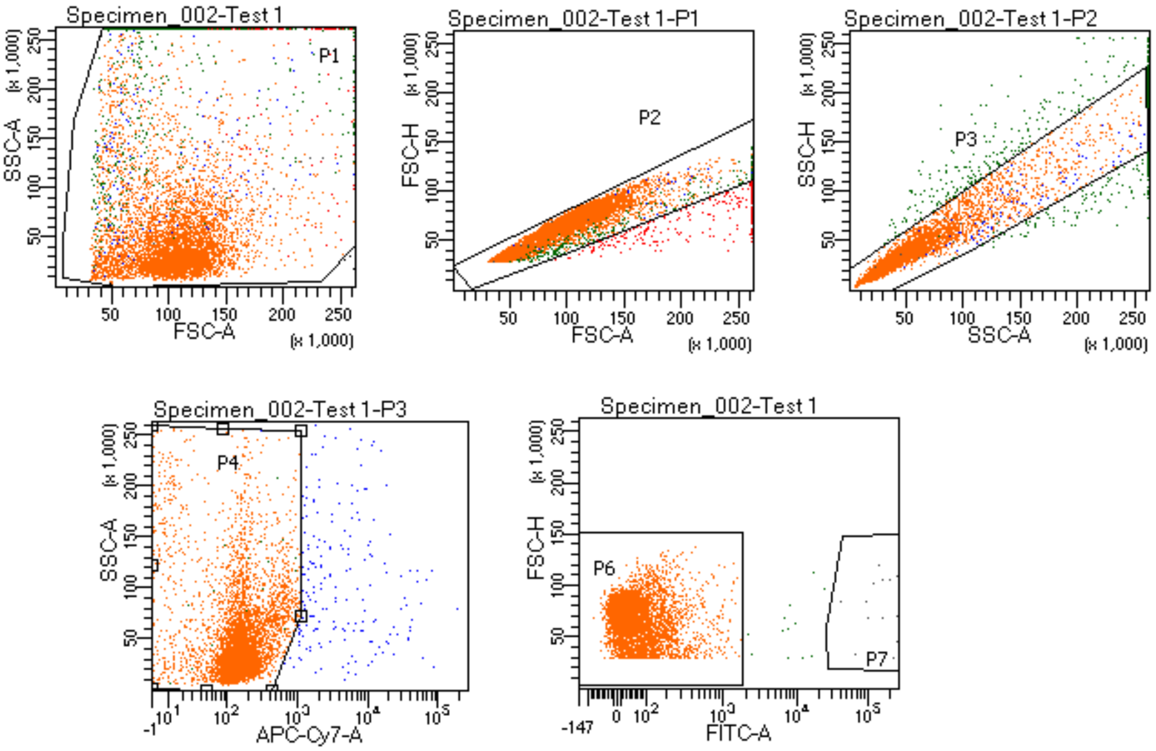

Tube: Test 1

Population	#Events	%Parent	%Total
All Events	6,279	####	100.0
P1	6,273	99.9	99.9
P2	6,019	96.0	95.9
P3	5,335	88.6	85.0
P4	5,141	96.4	81.9
P6	5,115	99.5	81.5
P7	14	0.3	0.2

BD FACSDiva 8.0.2

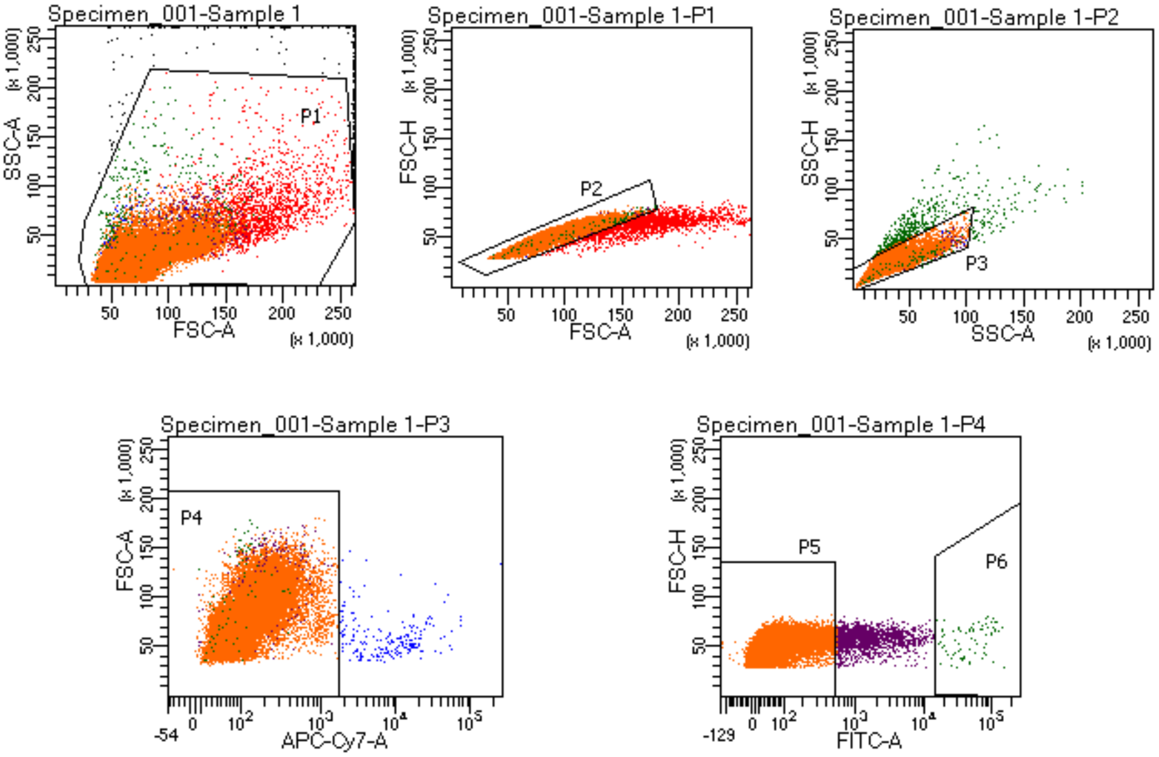

Tube: Sample 1

Population	#Events	%Parent	%Total
All Events	31,112	####	100.0
P1	30,819	99.1	99.1
P2	28,875	93.7	92.8
P3	28,380	98.3	91.2
P4	28,178	99.3	90.6
P5	26,502	94.1	85.2
P6	77	0.3	0.2

BD FACSDiva 8.0.2

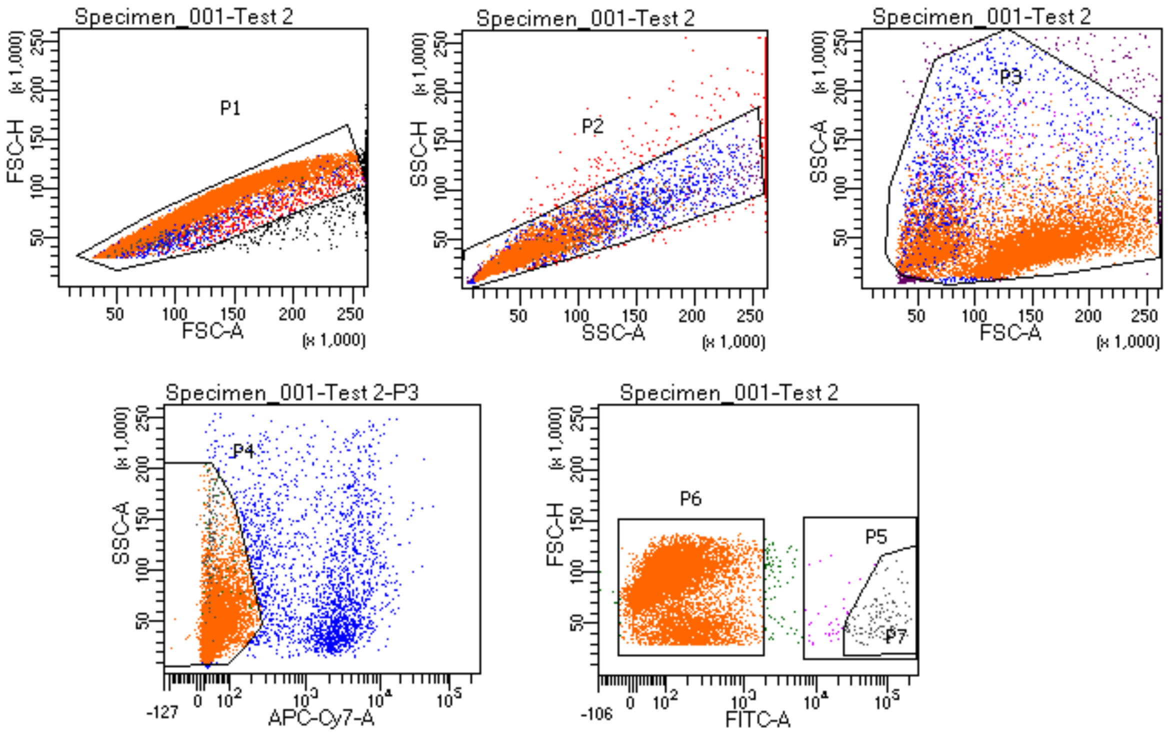

Tube: Test 2

Population	#Events	%Parent	%Total
All Events	14,894	####	100.0
P1	13,608	91.4	91.4
P2	12,761	93.8	85.7
P3	12,432	97.4	83.5
P4	10,136	81.5	68.1
P5	168	1.7	1.1
P6	9,879	97.5	66.3
P7	129	1.3	0.9

BD FACSDiva 8.0.2

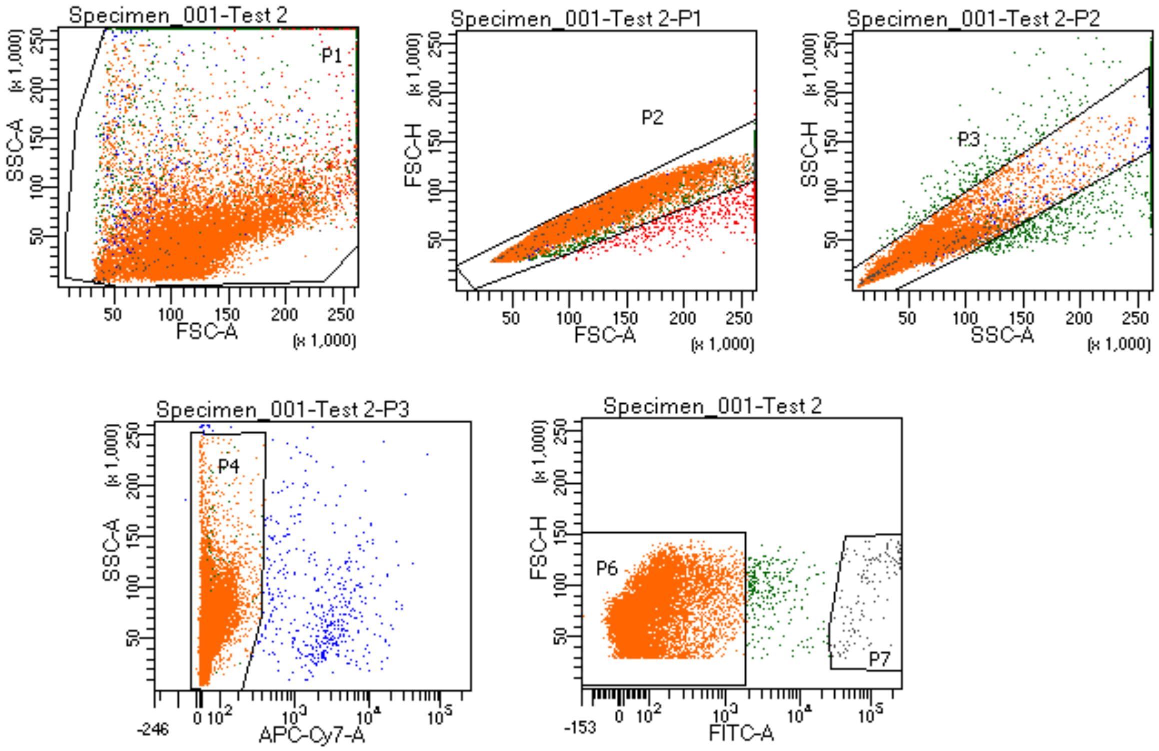

Tube: Test 2

Population	#Events	%Parent	%Total
All Events	15,783	####	100.0
P1	15,767	99.9	99.9
P2	15,061	95.5	95.4
P3	13,870	92.1	87.9
P4	13,379	96.5	84.8
P6	12,998	97.2	82.4
P7	136	1.0	0.9

BD FACSDiva 8.0.2

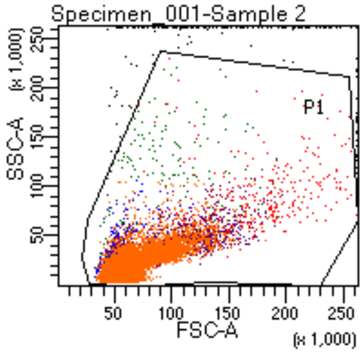

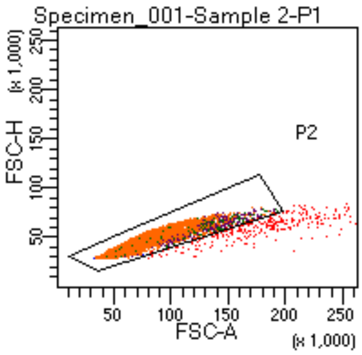

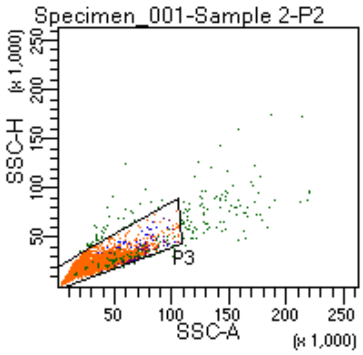

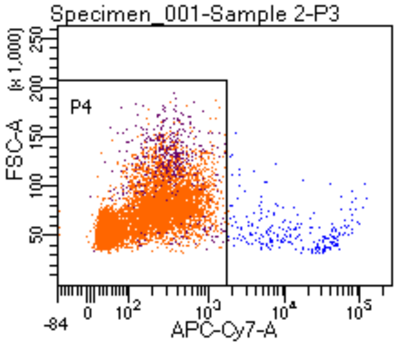

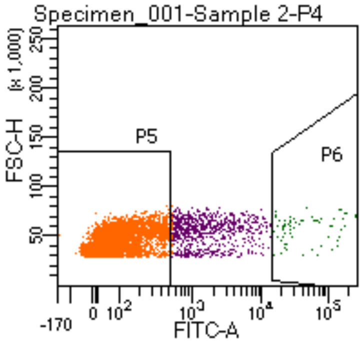

Tube: Sample 2

Population	#Events	%Parent	%Total
All Events	9,912	####	100.0
P1	9,698	97.8	97.8
P2	9,394	96.9	94.8
P3	9,231	98.3	93.1
P4	8,990	97.4	90.7
P5	8,145	90.6	82.2
P6	67	0.7	0.7

BD FACSDiva 8.0.2

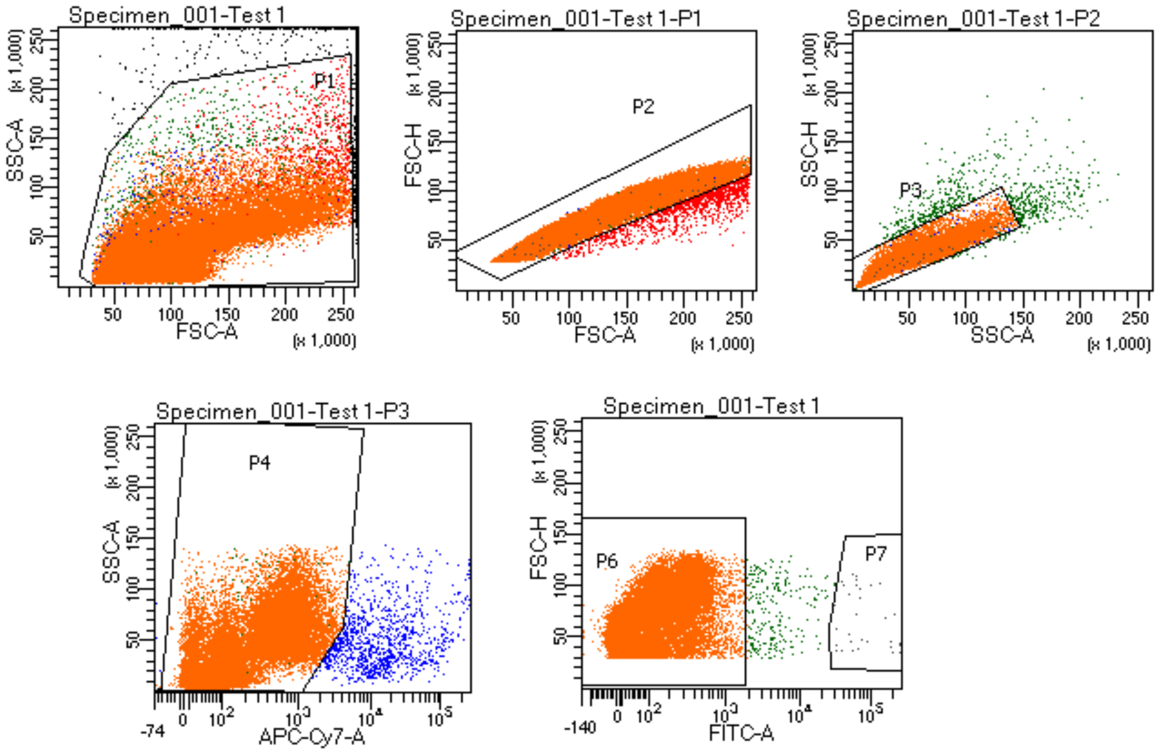

Tube: Test 1

Population	#Events	%Parent	%Total
All Events	35,855	####	100.0
P1	33,532	93.5	93.5
P2	32,089	95.7	89.5
P3	31,390	97.8	87.5
P4	30,246	96.4	84.4
P6	29,987	99.1	83.6
P7	31	0.1	0.1

BD FACSDiva 8.0.2

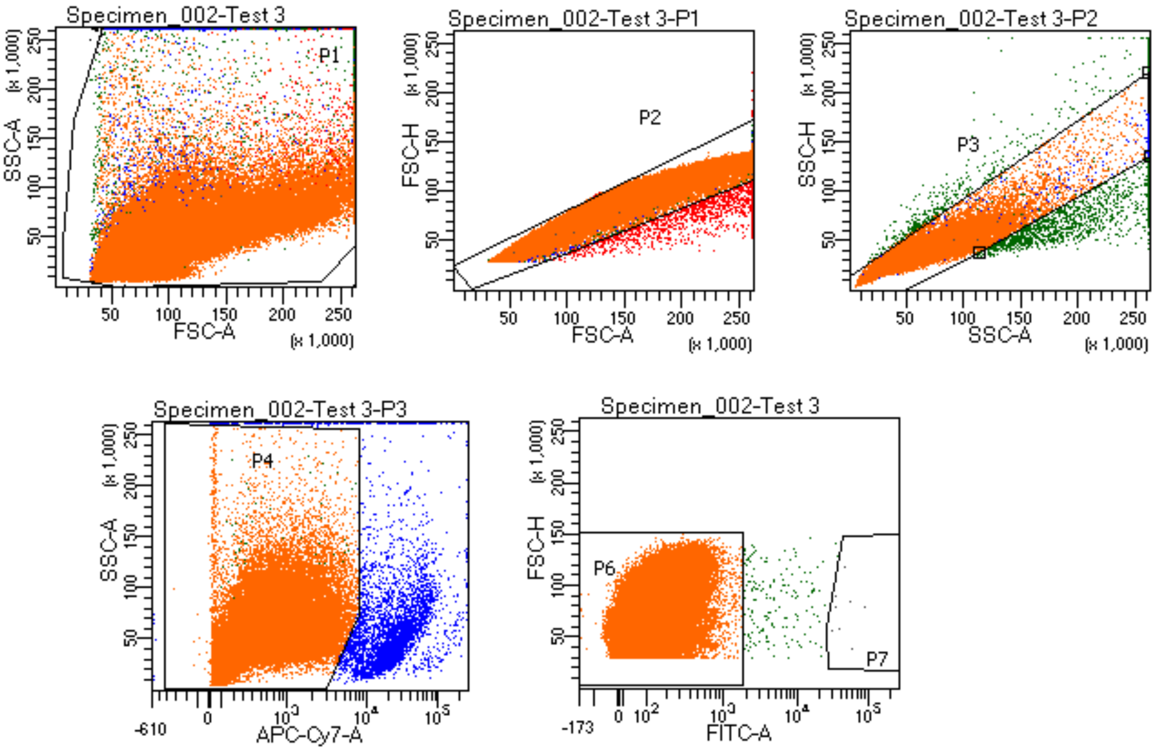

Tube: Test 3

Population	#Events	%Parent	%Total
All Events	76,665	####	100.0
P1	76,531	99.8	99.8
P2	74,608	97.5	97.3
P3	72,389	97.0	94.4
P4	68,312	94.4	89.1
P6	68,142	99.8	88.9
P7	9	0.0	0.0

BD FACSDiva 8.0.2

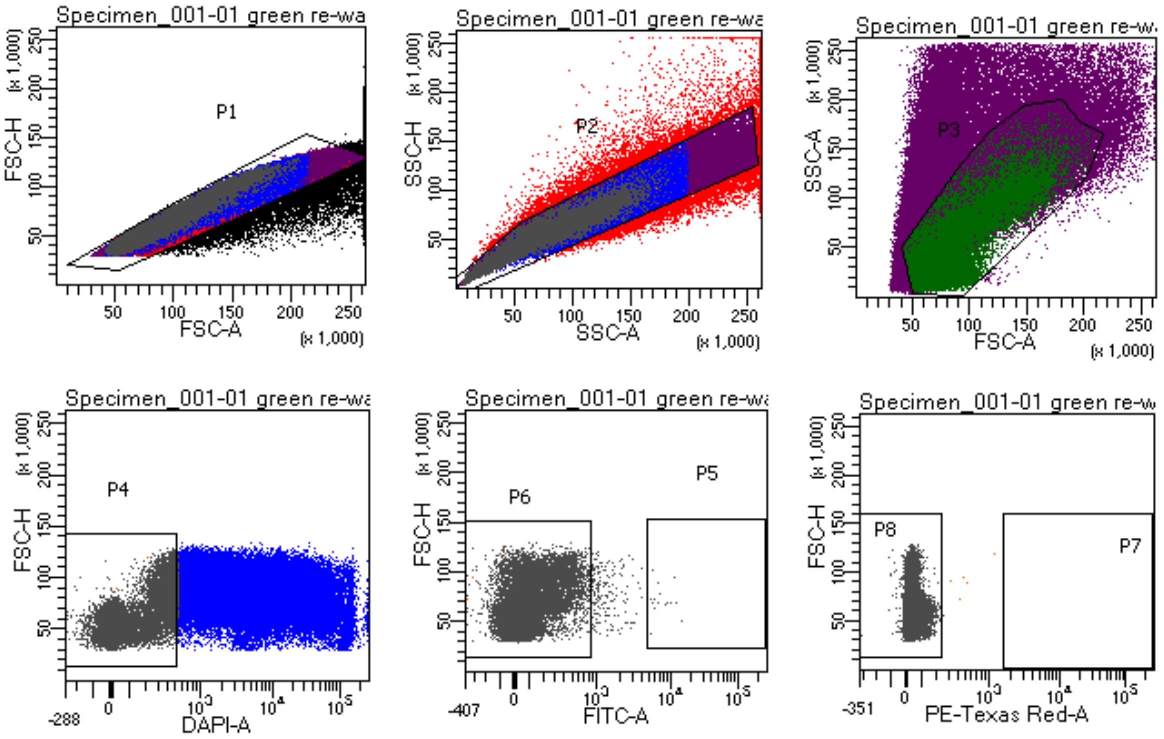

Tube: 01 green re-washed

Population	#Events	%Parent	%Total
All Events	146,861	####	100.0
P1	130,282	88.7	88.7
P2	107,536	82.5	73.2
P3	68,940	64.1	46.9
P4	14,691	21.3	10.0
P5	15	0.1	0.0
P6	14,518	98.8	9.9
P7	0	0.0	0.0
P8	14,686	100.0	10.0

BD FACSDiva 8.0.2

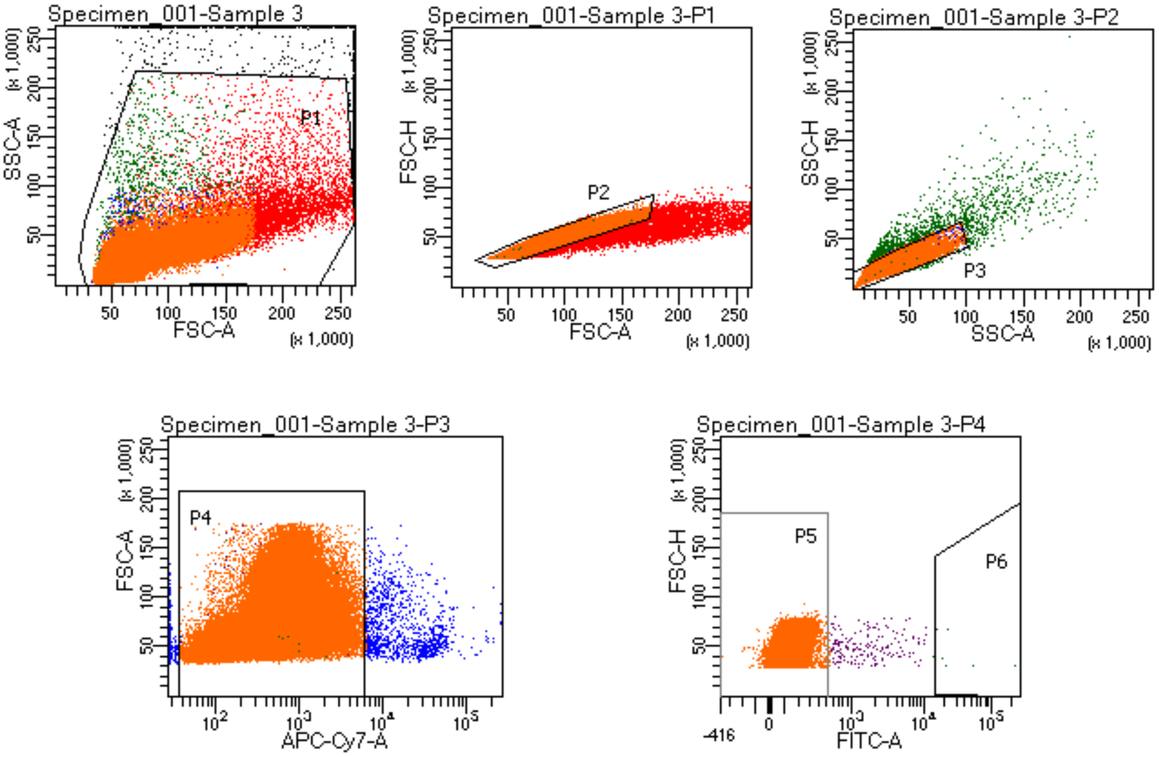

Tube: Sample 3

Population	#Events	%Parent	%Total
All Events	177,305	####	100.0
P1	175,831	99.2	99.2
P2	168,291	95.7	94.9
P3	166,913	99.2	94.1
P4	165,425	99.1	93.3
P5	165,248	99.9	93.2
P6	8	0.0	0.0

BD FACSDiva 8.0.2

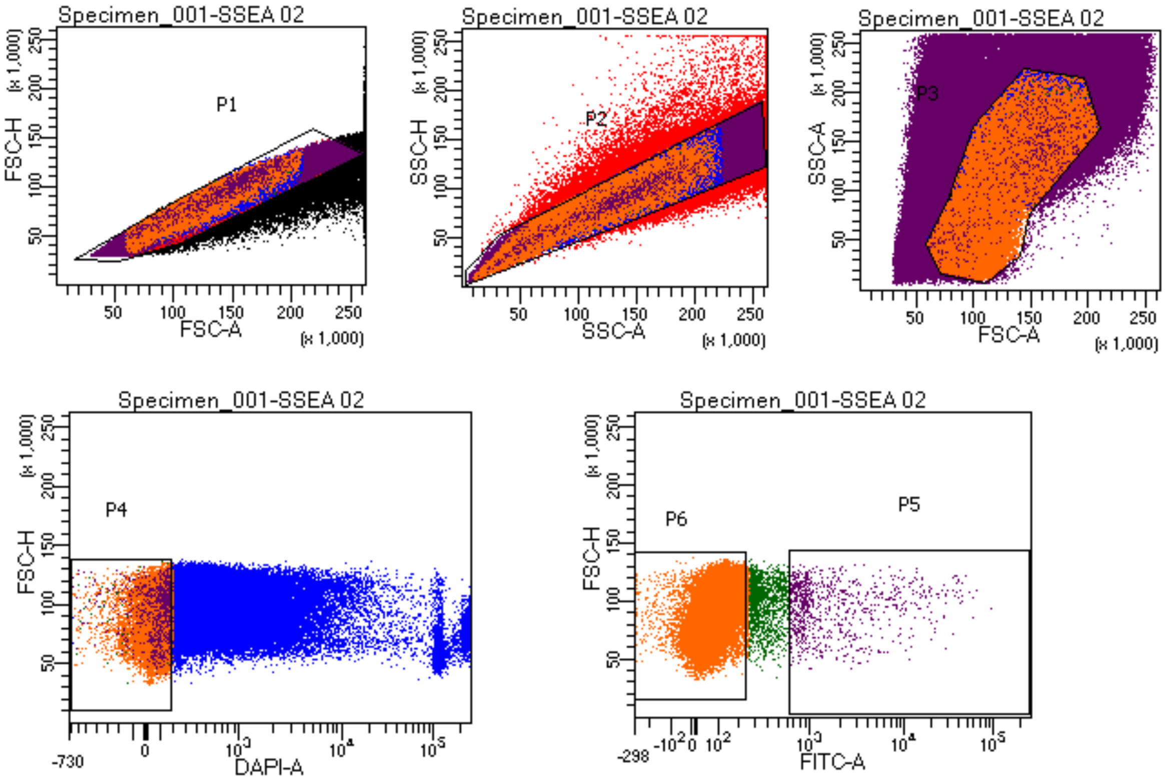

Tube: SSEA 02

Population	#Events	%Parent	%Total
All Events	369,615	####	100.0
P1	326,363	88.3	88.3
P2	279,809	85.7	75.7
P3	167,180	59.7	45.2
P4	45,421	27.2	12.3
P5	830	1.8	0.2
P6	43,344	95.4	11.7

BD FACSDiva 8.0.2

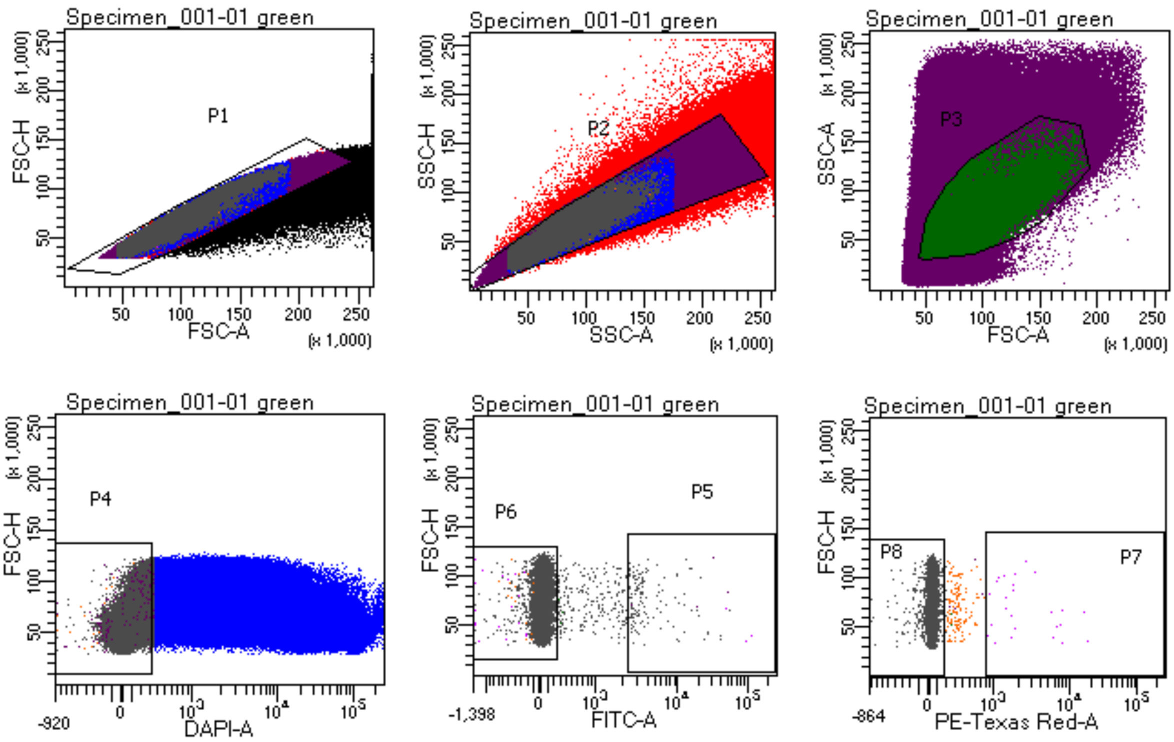

Tube: 01 green

Population	#Events	%Parent	%Total
All Events	657,117	####	100.0
P1	616,133	93.8	93.8
P2	505,859	82.1	77.0
P3	301,048	59.5	45.8
P4	36,146	12.0	5.5
P5	136	0.4	0.0
P6	35,802	99.0	5.4
P7	25	0.1	0.0
P8	35,976	99.5	5.5

BD FACSDiva 8.0.2

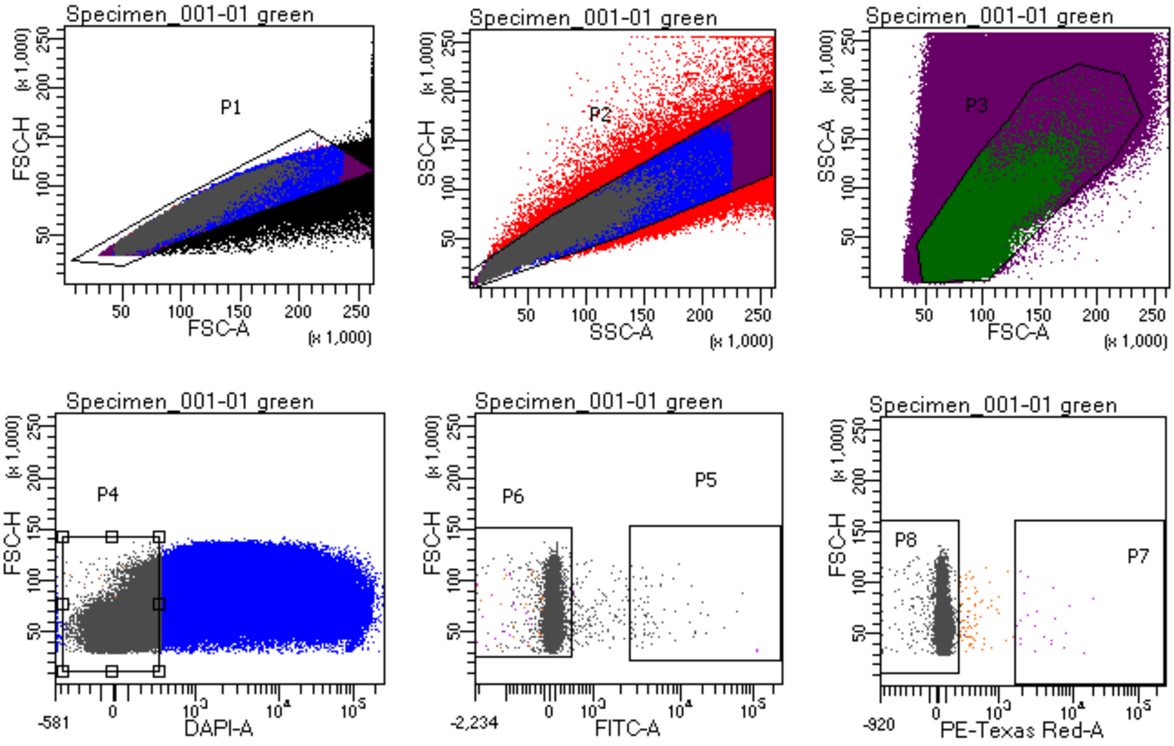

Tube: 01 green

Population	#Events	%Parent	%Total
All Events	787,466	####	100.0
P1	694,672	88.2	88.2
P2	617,015	88.8	78.4
P3	534,962	86.7	67.9
P4	53,702	10.0	6.8
P5	77	0.1	0.0
P6	53,497	99.6	6.8
P7	21	0.0	0.0
P8	53,605	99.8	6.8

BD FACSDiva 8.0.2

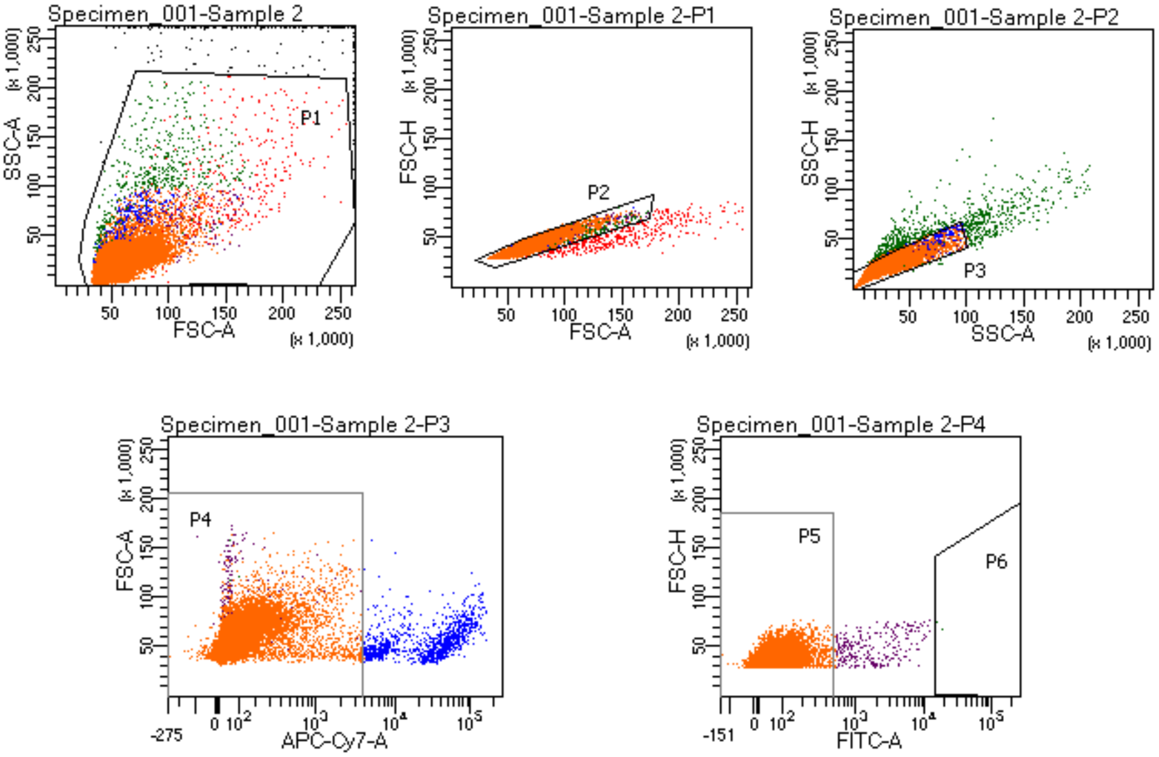

Tube: Sample 2

Population	#Events	%Parent	%Total
All Events	24,402	####	100.0
P1	24,033	98.5	98.5
P2	23,592	98.2	96.7
P3	22,753	96.4	93.2
P4	21,707	95.4	89.0
P5	21,444	98.8	87.9
P6	3	0.0	0.0

BD FACSDiva 8.0.2

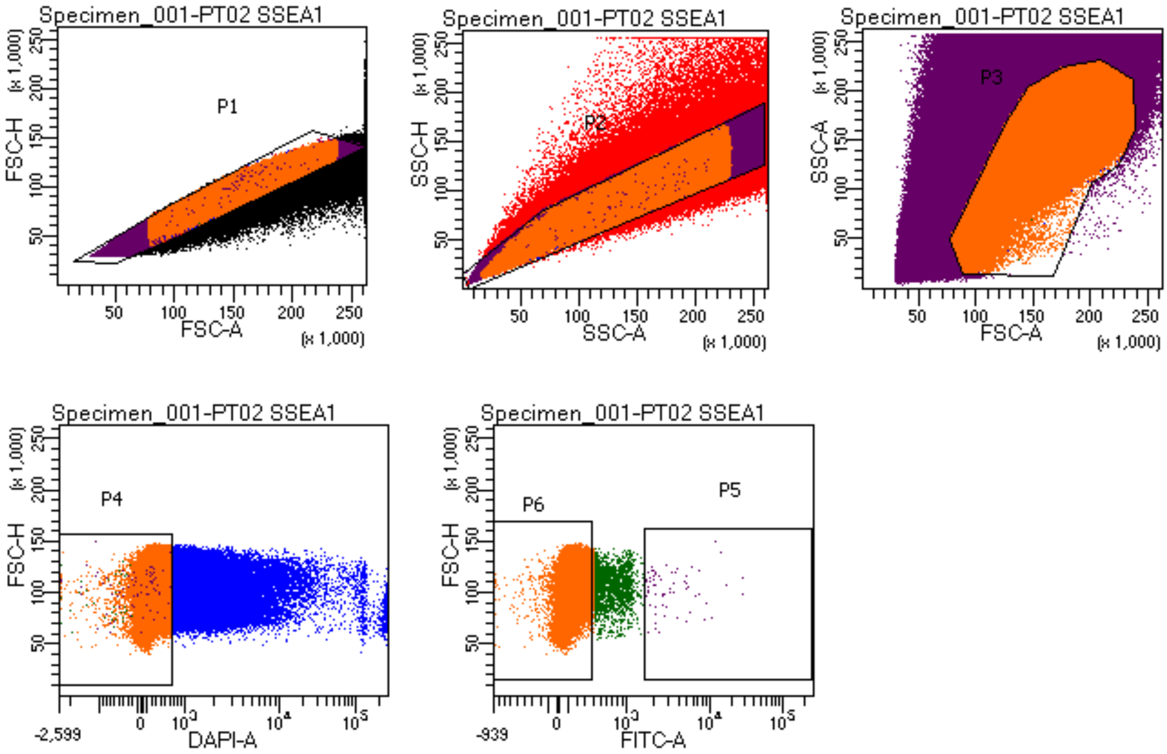

Tube: PT02 SSEA1

Population	#Events	%Parent	%Total
All Events	1,000,000	####	100.0
P1	883,327	88.3	88.3
P2	751,385	85.1	75.1
P3	444,833	59.2	44.5
P4	361,090	81.2	36.1
P5	71	0.0	0.0
P6	359,106	99.5	35.9

BD FACSDiva 8.0.2

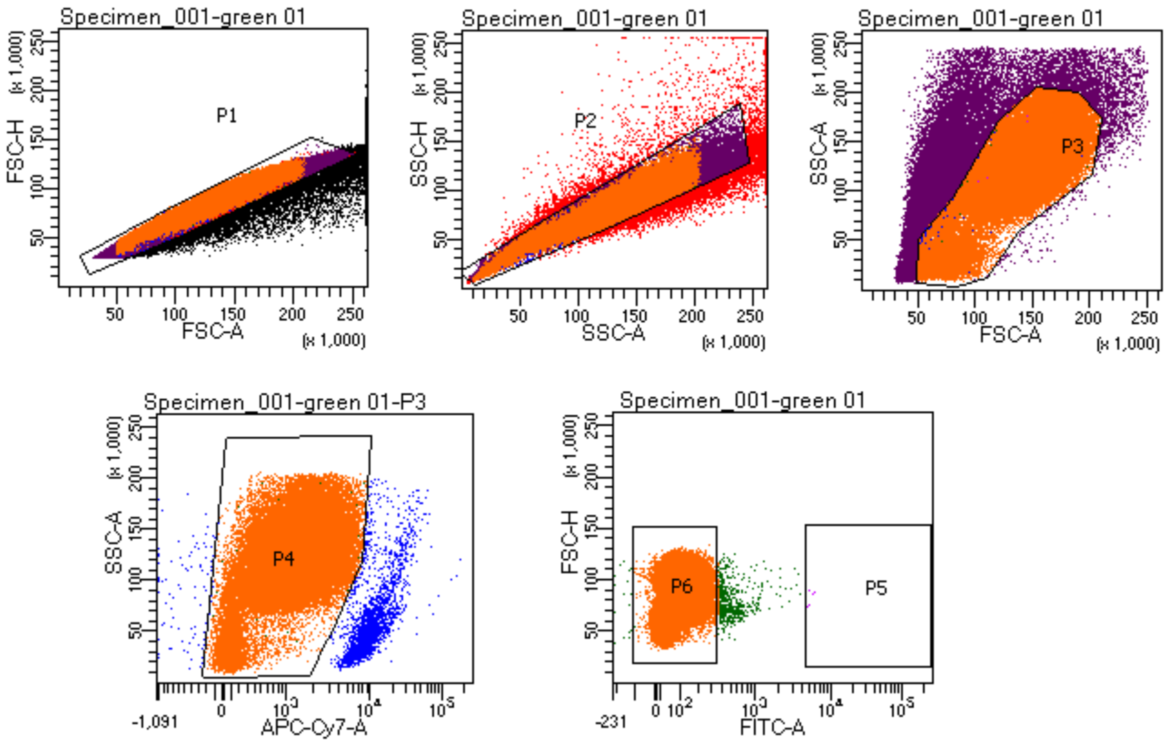

Tube: green 01

Population	#Events	%Parent	%Total
All Events	100,000	####	100.0
P1	85,873	85.9	85.9
P2	76,170	88.7	76.2
P3	51,325	67.4	51.3
P4	49,138	95.7	49.1
P5	5	0.0	0.0
P6	48,615	98.9	48.6

BD FACSDiva 8.0.2

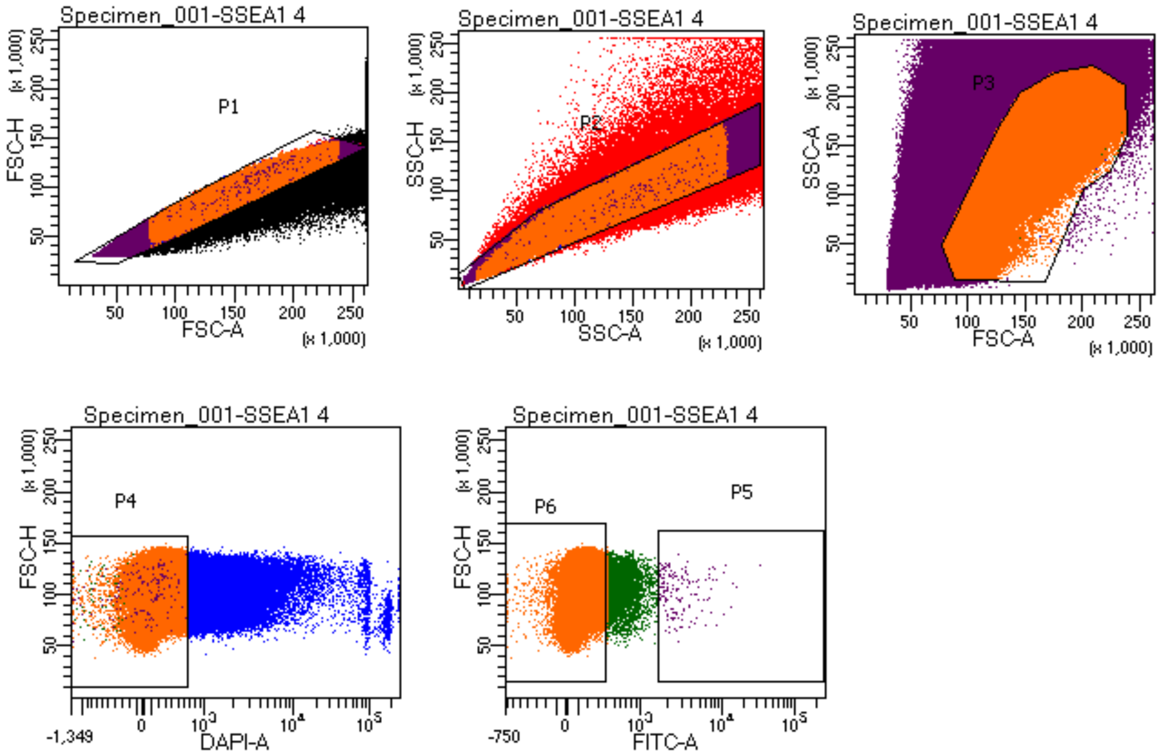

Tube: SSEA1 4

Population	#Events	%Parent	%Total
All Events	1,000,000	####	100.0
P1	842,214	84.2	84.2
P2	710,988	84.4	71.1
P3	415,582	58.5	41.6
P4	370,898	89.2	37.1
P5	166	0.0	0.0
P6	365,760	98.6	36.6
